# Highly accurate skin-specific methylome analysis algorithm as a platform to screen and validate therapeutics for healthy aging

**DOI:** 10.1186/s13148-020-00899-1

**Published:** 2020-07-13

**Authors:** Mariana Boroni, Alessandra Zonari, Carolina Reis de Oliveira, Kallie Alkatib, Edgar Andres Ochoa Cruz, Lear E. Brace, Juliana Lott de Carvalho

**Affiliations:** 1 Bioinformatics and Computational Biology Lab, Division of Experimental and Translational Research, Brazilian National Cancer Institute, Rio de Janeiro, RJ 20231-050 Brazil; 2 OneSkin Technologies, San Francisco, USA; 3 Genomic Sciences and Biotechnology Program, Catholic University of Brasilia, Brasilia, Brazil; 4 Faculty of Medicine, University of Brasilia, Brasilia, Brazil

**Keywords:** Aging, Skin aging, Epigenetics, DNA methylation, Fibroblasts, Molecular clock, DNAm age algorithm

## Abstract

**Background:**

DNA methylation (DNAm) age constitutes a powerful tool to assess the molecular age and overall health status of biological samples. Recently, it has been shown that tissue-specific DNAm age predictors may present superior performance compared to the pan- or multi-tissue counterparts. The skin is the largest organ in the body and bears important roles, such as body temperature control, barrier function, and protection from external insults. As a consequence of the constant and intimate interaction between the skin and the environment, current DNAm estimators, routinely trained using internal tissues which are influenced by other stimuli, are mostly inadequate to accurately predict skin DNAm age.

**Results:**

In the present study, we developed a highly accurate skin-specific DNAm age predictor, using DNAm data obtained from 508 human skin samples. Based on the analysis of 2,266 CpG sites, we accurately calculated the DNAm age of cultured skin cells and human skin biopsies. Age estimation was sensitive to the biological age of the donor, cell passage, skin disease status, as well as treatment with senotherapeutic drugs.

**Conclusions:**

This highly accurate skin-specific DNAm age predictor constitutes a holistic tool that will be of great use in the analysis of human skin health status/molecular aging, as well as in the analysis of the potential of established and novel compounds to alter DNAm age.

## Background

Aging is defined as a complex, multifactorial process associated with functional decline of organs and tissues, leading to increased chances of death [1]. Currently, genomic instability, telomere attrition, epigenetic alterations, loss of proteostasis, deregulated nutrient sensing, mitochondrial dysfunction, cellular senescence, stem cell exhaustion, and altered intercellular communication are considered the hallmarks of aging, emerging as active areas of investigation [1]. Age-related epigenetic alterations have gained prominence in this context since the observation that DNA methylation (DNAm) undergoes predictable time-dependent modifications, which can be explored as a highly accurate method to estimate the molecular versus the chronological age of human tissues [2].

Recently, additional factors beyond time have been shown to influence DNAm age, such as genetic diseases [3, 4], infectious diseases [5], inflammatory disorders [6, 7], and lifestyle factors, such as exercise and obesity [8], to cite a few. DNAm data was also significantly correlated to mortality risk [9]. Therefore, DNAm has evolved from a chronological age estimation tool to an indicator of overall health.

Cellular senescence is an evolving concept and is currently defined as a cellular state characterized by four main aspects: an irreversible cell-cycle arrest, an inflammatory senescence-associated secretory phenotype (SASP), macromolecular damage (DNA, protein and lipid damage), and altered metabolism [10]. Other aspects attributed to senescent cells include apoptosis resistance, senescence-associated heterochromatin foci, and morphological alterations. Even though cellular senescence and organismal aging are not synonymous, recent data supports the fact that DNAm aging observed in vivo also occurs in vitro in senescent cell cultures (e.g., mammalian cell cultures) in a highly reproducible pattern [11, 12]. Indeed, DNAm of cultured cells can be used to predict cellular passage, regardless of donor chronological age [13]. In this context, even though the mechanism by which cellular passage and time promote DNAm alterations in cells/tissues has not been clarified, a few functional correlations between DNA methylation and phenotype have been described. For instance, as shown by Xie et al. [14], during replicative cellular senescence, hypermethylation occurs mainly in promoter sites of genes responsible for cellular biosynthesis and metabolism regulation, promoting, or at least favoring, a gradual decrease of biosynthetic processes observed in senescent cells. Importantly, not only epigenetic alterations are shared between cellular senescence and organismal aging but also other molecular and phenotypic aspects, including telomere attrition and slower renewal rate [2]. These findings fuel the current perception that cellular senescence is a good model to be investigated in aging studies. The possibility to study aging in vitro initiates the opportunity to apply DNAm age as a parameter to screen and/or validate potential investigational senotherapeutic compounds, defined as molecules targeting senescent cells, either by promoting their death (senolytics), or altering the senescence status (senomorphics) [15].

DNAm aging has been characterized in numerous tissues, including the skin [16]. Nevertheless, perhaps due to the high influence of environmental factors on skin aging [17], the pan-tissue algorithm developed to estimate tissue aging failed to accurately calculate the chronological age of cultured fibroblasts [2]. Also, phenotypic skin analysis failed to relate to blood DNAm age [18]. In recognition of the limited accuracy of the pan-tissue estimator of DNAm age, Horvath et al. developed a skin and blood clock [19]. In the present study, we developed a skin-specific algorithm that calculates DNAm age of skin samples with high accuracy and a low error compared to existing molecular age estimators. The present algorithm can be used in a scalable platform to validate the effect of new and established compounds to the skin DNAm age.

## Results

### Dataset description

The utilized workflow is depicted in Additional File 1–Fig S1. For the algorithm development, we analyzed previously published DNAm data of human skin biopsies, retrieved from three datasets. The specific datasets, GSE51954, E-MTAB-4385, GSE90124, are available in public databanks (GEO and ArrayExpress) and each comprise epigenetic data, as well as additional information about the analyzed samples, i.e., origin tissue, donor sex, and chronological age (18–95 years old). A total of 508 samples (40 derived from the dermis, 146 from the epidermis, and 322 samples derived from whole skin tissue) were interrogated for whole-genome methylation levels by more than 450,000 CpG probes per sample. The main characteristics of the cohort are described in Additional File 2–Supplementary Table S1.

### Data normalization and pre-selection of features

Each dataset was individually processed for quality control and merged for preprocessing in order to build a machine learning algorithm able to accurately predict DNAm age. After normalizing all datasets by quantile (Additional File 3–Fig S2), we obtained a homogeneous dataset with 397,598 probes.

We then removed 1720 cross-reactive probes [20] and 26,490 probes which were not present in the new version of the EPIC array. Probes targeting sex chromosomes were also removed, resulting in 369,388 probes. A feature selection step was performed to reduce the dimensionality of our dataset using a package as a wrapper for three different algorithms implementations (Additional File 4–Fig S3). Since the number of features is much greater than the samples in our dataset (the curse of dimensionality), this is an important step to reduce a model’s overfitting, i.e., highly accurate on training data but poor generalization on unseen test data, while improving its accuracy if a proper feature' subset is chosen [21]. By reducing the complexity of a model, we also reduce the time of training. Each different algorithm ranked the 369,388 probes according to their importance to predict the sample’s age. The wrapper also ranked the probes according to the union-importance, which was calculated considering the results of the three algorithms together. We first retrieved the top 100 probes ranked according to each different algorithm and the top 2000 probes ranked according to the union-importance. We also added-up the top 400 probes most correlated with age according to Pearson’s correlation coefficient, totalizing 2410 probes (some probes were ranked by more than one strategy). Next, we removed features that are correlated with the response, but highly correlated with each other. The final dataset consisted of 2266 probes, divided into training (249 samples) and testing (259 samples) data subsets. Samples were randomly selected for training and testing datasets following a balanced distribution between the donor ages (cut-off of 5 samples per age window, wherein an age window is approximately 7 years) (Table 1, Additional File 1–Fig S1 and Additional File 5–Fig S4), in order to avoid overfitting in older ages, since the full dataset was enriched in older donor samples. Dermis samples were all placed in the training dataset, due to their small number.

**Table 1:** Training and testing data description

Dataset	Number of samples	Type of sample	Sex	Ethnicity	Age
Training	249	40 dermis 99 epidermis 110 whole skin	214 F 35 M	Caucasian	Min. 18.00 1st Qu. 35.70 Median 53.37 Mean 51.56 3rd Qu. 66.21 Max. 95.00
Testing	259	0 dermis 47 epidermis 212 whole skin	259 F 0 M	Caucasian	Min. 20.00 1st Qu. 54.59 Median 62.46 Mean 59.38 3rd Qu. 67.67 Max. 74.97

### Selection of the best skin-specific DNAm age predictor

Next, we tested five machine learning (ML) algorithms to build different models and select the best skin-specific DNAm age predictor, including the random forest; support vector machines (SVMs); ridge regression, which penalizes the size of parameter estimates by shrinking them toward zero (L1 penalty) in order to decrease model complexity while keeping all variables in the model; Lasso (least absolute shrinkage and selection operator), which drops some features by penalizing coefficients and driving them to zero (L2 penalty); and elastic net regression, a regularized regression method that linearly combines the L1 and L2 penalties of the Lasso and ridge methods. After training, an optimal regression was selected based on a minimum mean absolute error (MAE) and root mean squared error (RMSE), and maximum *R*^2^ (Additional File 6–Fig S5). Ridge and elastic net displayed similar performances, and due to its characteristics, elastic net was chosen. After the 50-fold cross-validation, the best model was obtained with fraction = 1 and lambda = 1 × 10^−4^, corresponding to a regression model with an *R*^2^ of 0.99, RMSE of 2.34 years, and MAE of 1.94 years (Additional File 6–Fig S5).

**Fig. 1:**
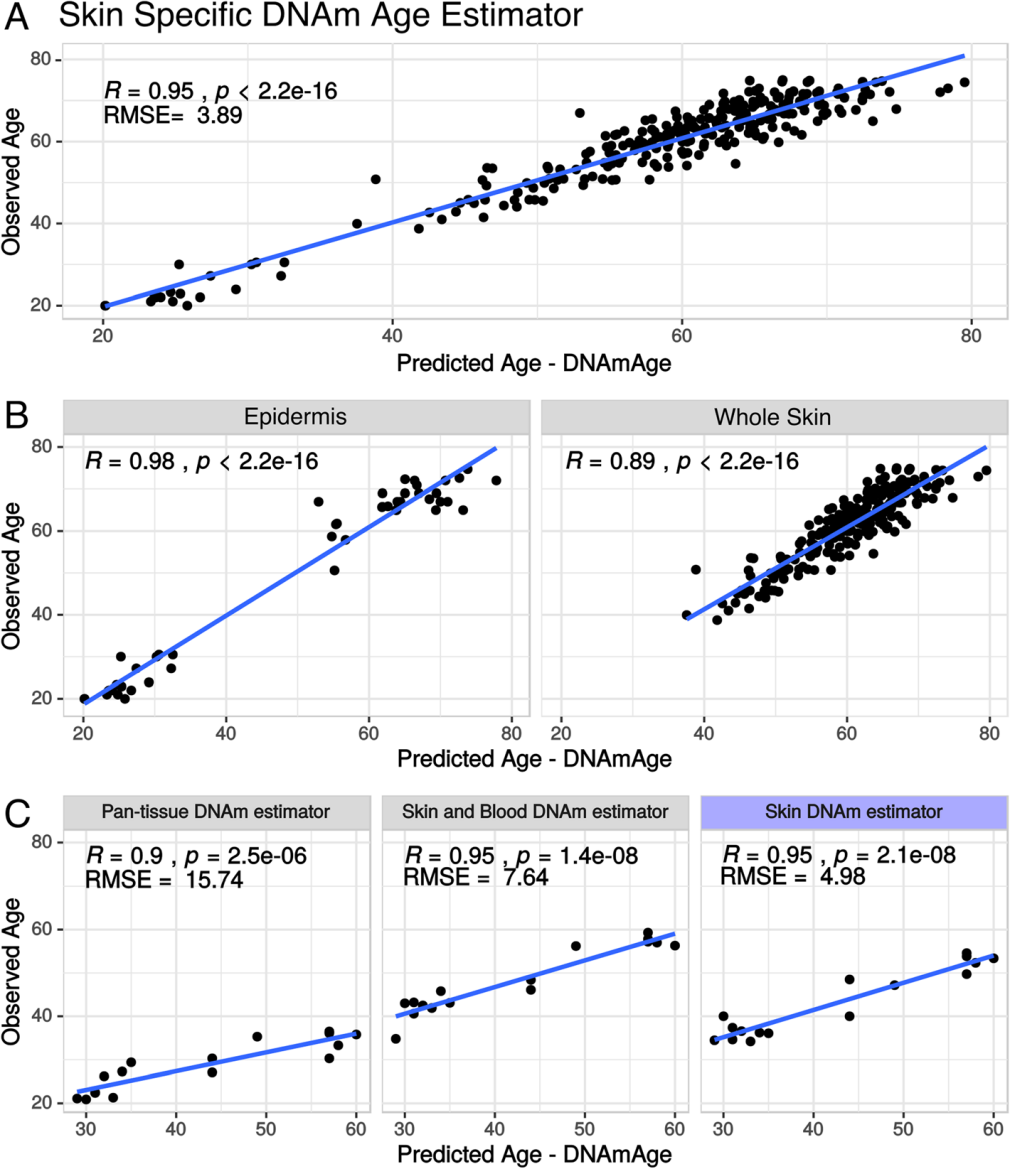
Age estimation accuracy of the Skin-Specific DNAm age predictor. **a** Correlation analysis between predicted age using the elastic net model and chronological age for all samples from the testing dataset. **b** A correlation was evaluated considering only epidermal or whole skin samples from the testing dataset. **c** Performance comparison with previously published algorithms by a correlation analysis between predicted and chronological age using a novel dataset of whole skin biopsies (external validation)

The elastic net model was able to predict the testing dataset with high confidence. The correlation between predicted and chronological age was 0.95 (*p* ≤ 2.2 × 10^−16^) with an RMSE of 3.89 years (Fig. 1a). When comparing algorithm performance between epidermal and whole skin methylome data, a slightly improved accuracy was observed for epidermis samples (Fig. 1b).

Although the machine learning step did not utilize the testing dataset during training, this dataset was a subset of the original dataset used for training the model. We then evaluated the accuracy of the model using a completely independent new subset of 16 whole skin biopsies that include a methylation profile accessed using the EPIC array. By using this external dataset, we again obtained highly accurate predictions, with a correlation between predicted and chronological age of 0.95 (*p* ≤ 2.1 × 10^−8^) and an RMSE of 4.98 years, outperforming previous DNAm estimators described in the literature (Fig. 1c and Additional File 7—Supplementary Table 2) [2, 19].

### Predictors as skin aging biomarkers

In order to find potential new biomarkers for skin aging and skin age-reversal interventions, we next evaluated the probes interrogated in our model and the genes in which they are associated. From the 2266 probes, 53% were positively correlated with age in the final model. Most probes located within the body of gene sequence (34.5%), 11.5% were localized on the 1stExon, 3.4% on the 3′UTR, 14.6% on the 5′UTR, 20.3% on the TSS1500, and 15.6% on the TSS200.

**Fig. 2:**
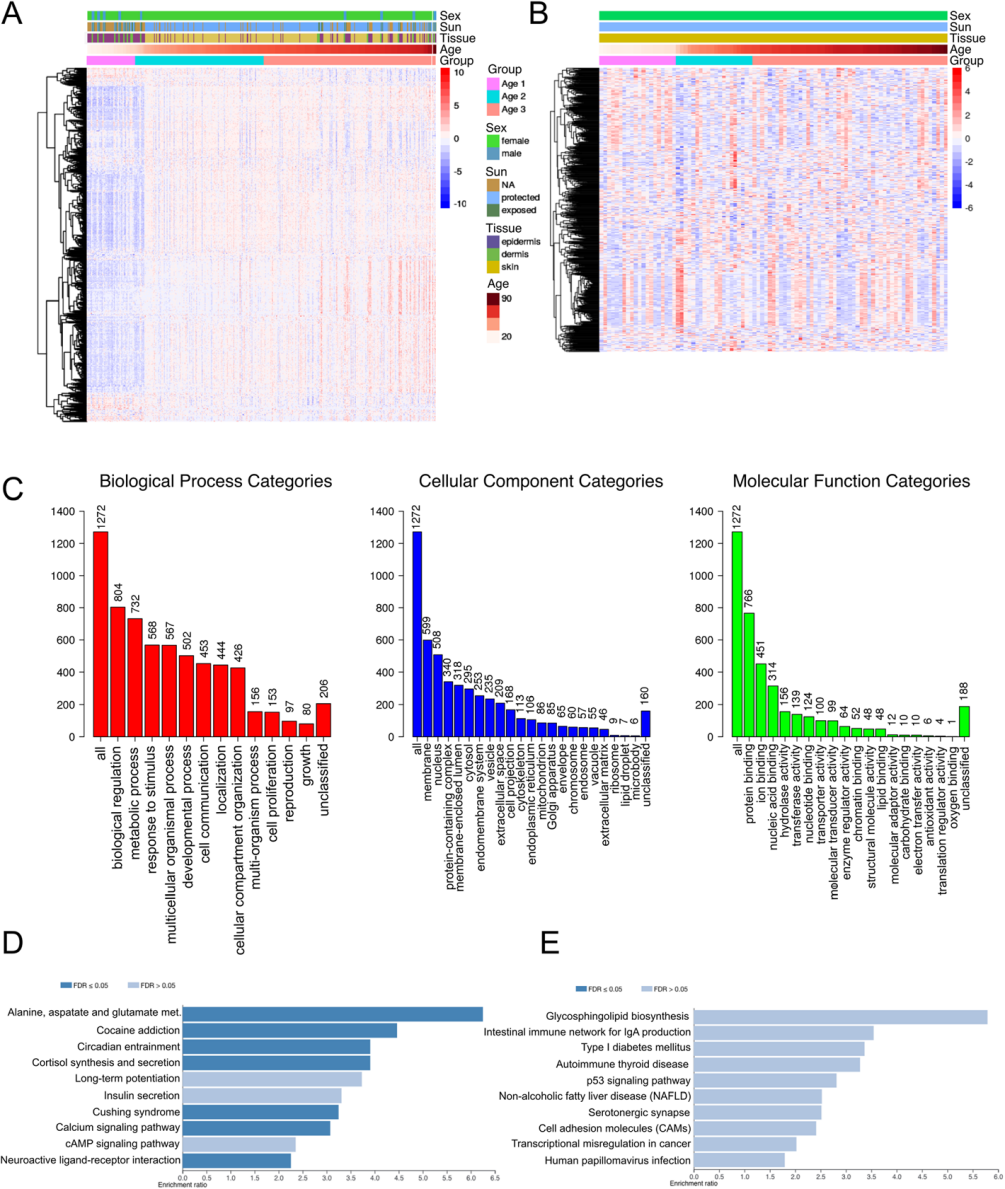
Effects of aging on CpGs and genes associated with the skin-specific DNAm age predictor. **a** Heat map of DNA methylation levels of probes associated with the model across all samples. Only probes with a SD between the second and third quartile are plotted. Color codes represent beta DNAm values after row-wise z-score transformation. Probes (rows) were clustered using Pearson correlation. Samples were ordered according to age. Features regarding tissue of origin, sun exposure, sex, and age group (age 1: < 30 years old, age 2: between 30 and 60 years old, and age 3: > 60 years old) are also shown. **b** Heat map of CpG-related genes expression levels associated with the model across all samples. Only genes with a SD higher than the second quartile are plotted. Color-codes represent log(normalized expression + 1) values after row-wise z-score transformation. Genes (rows) were clustered using Pearson correlation. Samples were ordered as shown in (**a**). **c** Gene ontology (GO) enrichment summary for genes associated with probes in the model. **d** Over representation analysis using KEGG database genes associated with probes positively correlated with age and **e** genes associated with probes negatively correlated with age. Dark bars represent significantly enriched pathways after controlling for false discovery rate (FDR) using the Bonferroni method

In general, the methylation level differences of probes used in our model were strongly influenced by tissue type (i.e., epidermis, dermis, or whole skin) and sun exposure (ultraviolet radiation (UV) exposure). Even though the methylation level differences across different ages were relatively small, a large drift was observed around age 30, where some probes displayed increased methylation levels (Fig. 2a). According to the Illumina array manifest, the 2266 probes selected could be related to 1572 unique genes. From those, 50% of genes were associated with positively correlated probes and 58% had probes selected in their promoter region. We also compared the expression alterations across aging of probe-associated genes, using an independent publicly available RNA-Seq dataset composed of 91 skin biopsy samples obtained from sun-protected regions (inner arm) of donors ranging from 19 to 89 years old. When evaluating gene expression alteration across ages, a less noticeable correlation between probes-associated gene expression and aging could be observed (Fig. 2b).

When evaluating the pathway enrichment within all probe-associated genes, only 1272 gene IDs were unambiguously mapped to unique Entrez Gene IDs. Gene ontology analysis determined that approximately 39% of genes were associated with developmental processes, 36% with cell communication, and 25% with functions related to nucleic acid binding (Fig. 2c). Genes associated with positively correlated probes were significantly enriched in pathways such as calcium signaling and cortisol synthesis and secretion (false discovery rate (FDR) < 0.05, Bonferroni test, Fig. 2d and Additional File 8–Supplementary Table 3), while no significant results were obtained for genes associated with negatively correlated probes after controlling for FDR (Fig. 2e).

**Fig. 3:**
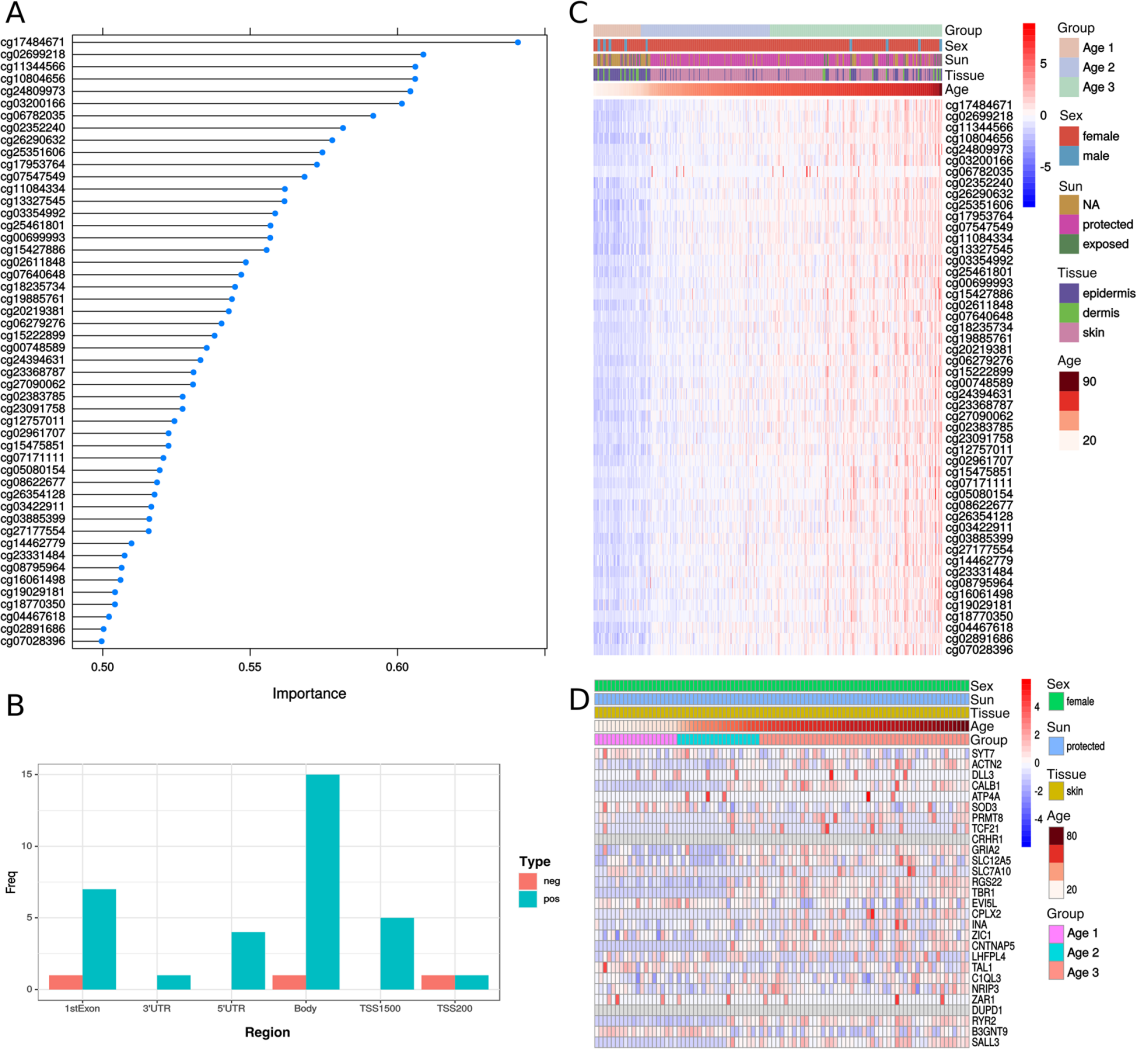
Importance of predictors. **a** Variable importance for top 50 predictors according to the Loess r-squared variable importance given by the varImp function from caret R package. **b** Frequency of regions where top 50 probes are located. Blue color refers to probes positively correlated with age in the model, and red color refers to probes negatively correlated with age. **c** Heat map of DNA methylation levels of the top 50 probes. Color codes represent beta DNAm values after row-wise z-score transformation. Probes (rows) are ordered according to their importance. Samples were ordered according to their age. **d** Heat map of the top 50 CpG-related gene expression levels associated with the model across all samples. Only genes with SD higher than the second quartile are plotted. Color-codes represent log(normalized expression + 1) values after row-wise z-score transformation. Genes (rows) were clustered using Pearson correlation. Samples were ordered according to their age. Features regarding tissue of origin, sun exposure, sex, and aging group (age 1: under 30 years old, age 2: between 30 and 60 years old and age 3: over 60 years old) are also shown

We next evaluated the importance of each probe, considering their contribution to the model (Loess r-squared variable importance). The top 50 most important probes are highlighted in Fig. 3a. The majority of these probes were located in the gene body (44%) (Fig. 3b) and positively correlated with age (92%) (Fig. 3c). However, only a few probe-associated genes had their mRNA expression levels correlated with age such as *GRIA2*, *TBR1*, *RGS22* (positively correlated), and *B3GNT9* (negatively correlated) (Fig. 3d). When considering the genes associated with the top 300 most important probes for pathways enrichment analysis, cAMP signaling and neuroactive ligand-receptor pathways were both enriched in our dataset (FDR < 0.05, Bonferroni, Table 2).

**Table 2:** Pathway enrichment results for genes associated with the top 300 key probes belonging to the skin-specific DNAm age predictor. The top 10 results are shown. The enrichment method ORA was performed using the KEGG database. *P* values were corrected to control for FDR using the Bonferroni method, statistically significant enrichment is highlighted in bold letters

Description	Enrichment ratio	*p* value	FDR	Genes
**cAMP signaling pathway**	**5**.**7451**	**2**.**1** × **10**^−**5**^	**6**.**7** × **10**^−**3**^	***ACOX3***; ***CACNA1C***; ***CNGA3***; ***GHSR***; ***GRIA2***; ***GRIN2D***; ***HTR1E***; ***PDE4C***; ***RYR2***
**Neuroactive ligand-receptor interaction**	**4**.**2918**	**2**.**0** × **10**^−**4**^	**3**.**2** × **10**^−**2**^	***AVPR1A***; ***CRHR1***; ***GHSR***; ***GRIA2***; ***GRIN2D***; ***HTR1E***; ***LEP***; ***PRLHR***; ***PTGFR***
Circadian entrainment	6.4865	9.6 × 10^−4^	0.104	*CACNA1C*; *CACNA1G*; *GRIA2*; *GRIN2D*; *RYR2*
Cardiac muscle contraction	7.0969	2.3 × 10^−3^	0.151	*CACNA1C*; *CACNG7*; *COX5A*; *RYR2*
Arrhythmogenic right ventricular cardiomyopathy (ARVC)	6.9941	2.4 × 10^−3^	0.151	*ACTN2*; *CACNA1C*; *CACNG7*; *RYR2*
Calcium signaling pathway	4.2087	2.8 × 10^−3^	0.151	*AVPR1A*; *CACNA1C*; *CACNA1G*; *GRIN2D*; *PTGFR*; *RYR2*
GABAergic synapse	5.8144	4.8 × 10^−3^	0.220	*ABAT*; *CACNA1C*; *GAD1*; *SLC12A5*
Type II diabetes mellitus	8.226	5.6 × 10^−3^	0.226	*CACNA1C*; *CACNA1G*; *IRS2*
Amyotrophic lateral sclerosis (ALS)	7.0969	8.4 × 10^−3^	0.303	*GRIA2*; *GRIN2D*; *NEFM*
Alcoholism	3.7009	1.1 × 10^−2^	0.350	*GRIN2D*; *H2AFY*; *HIST1H2AI*; *HIST1H2BK*; *HIST1H4I*

Finally, we analyzed for significant overlap between the probes interrogated by the skin-specific DNAm age predictor and both the pan-tissue and the skin and blood DNAm age estimators. As expected, the overlap between the skin-specific DNAm age predictor and the pan-tissue algorithm was negligible (14 of 2266 probes) (Additional File 9–Fig S6a). The number of common probes between the skin and blood DNAm age estimator and the newly developed skin-specific DNAm age predictor was 57. Eight probes were shared among the three predictors and are depicted in Additional File 10–Supplementary Table 4 and their methylation levels across age are shown in Additional File 9–Fig S6b. Shared probes were associated with eight genes, from which only six were present in our RNA-Seq dataset. When evaluating their mRNA expression, no differences among age groups were observed (Additional File 9–Fig S6c), showing that their contribution to the model is probably related to epigenetic changes during aging that do not influence the expression of the gene in which they are located or associated.

### Applications of the skin-specific DNAm age predictor

**Fig. 4:**
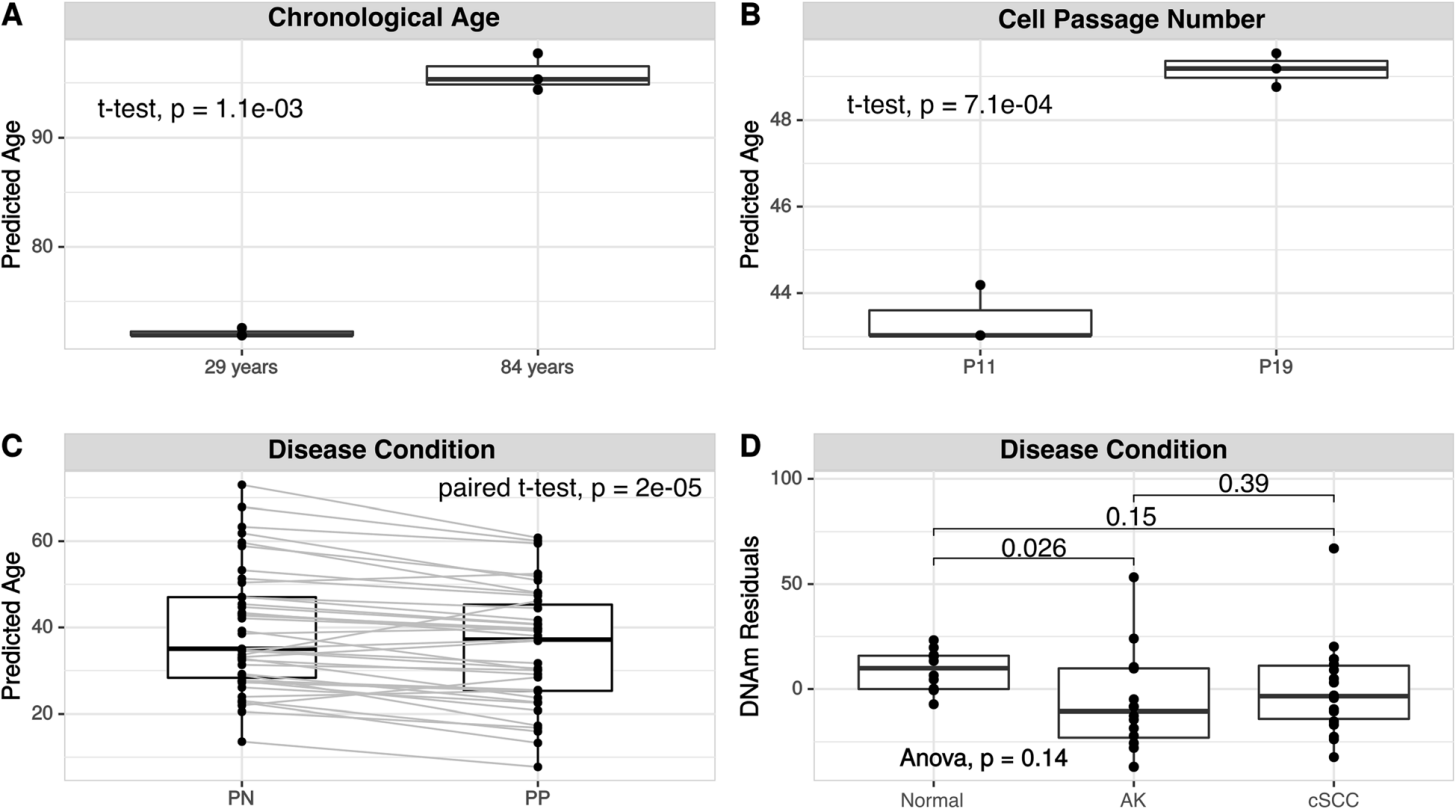
Skin-specific DNAm age predictor applications. **a** DNAm age of primary human dermal fibroblasts obtained from two healthy donors of different ages. **b** DNAm age of primary human dermal fibroblasts derived from an HGPS donor with different cell passage number. **c** DNAm age of human psoriatic (PP) and paired uninvolved psoriatic (PN) skin tissues (GSE73894). **d** DNAm age residuals of normal epidermis tissues, AK—actinic keratosis and cSCC—cutaneous squamous cell carcinoma epidermis samples (E-MTAB-5738)

Since the skin-specific algorithm developed here accurately estimates the DNAm age of skin samples, we decided to investigate whether the algorithm would be able to calculate the effect of different interventional-aging therapeutics over skin DNAm age. To do so, first, we verified the ability of our algorithm to predict DNAm age differences in primary human dermal fibroblasts obtained from donors of different chronological ages (Fig. 4a). While fibroblasts derived from a 29-year-old donor were predicted to have an average age of 72.4 (standard deviation—SD: 0.393), the average age of 96.4 (SD: 1.73) was calculated for fibroblasts derived from an 84-year-old donor (*p* = 0.001, *t* test, Fig. 4a), showing that our algorithm was able to accurately predict the progression of chronological age in fibroblasts of different donors. The fact that both cell preparations were analyzed at passage 22 explains, at least in part, the discrepancies between donor age and DNAm age-predicted for cultured cells. The capacity of our algorithm to detect the effect of cell passage was also confirmed using dermal fibroblasts isolated from a 6-year-old Hutchinson-Gilford Progeria (HGPS) patient. While cells at passage 11 presented a mean of 43.3 (SD: 0.673) years DNAm age, the same cell culture presented a mean DNAm age of 49.1 (SD: 0.39) years at passage 19 (*p* = 7.0 × 10^−4^, *t* test, Fig. 4b).

We also tested the capability of our algorithm to predict DNAm age alterations related to disease conditions such as psoriasis, a chronic inflammatory skin condition, cutaneous squamous cell carcinoma (cSCC), and UV-induced precancerous lesions termed actinic keratosis (AK). We predicted the molecular age of 39 samples from active psoriatic skin tissue (PP) and paired inactive control non-psoriatic skin tissues (PN) obtained from patients diagnosed with the disease (data obtained from [22]). Different from what has been previously shown [23], a statistically significant difference was observed between the DNAm age of PN (mean of 39.2 years, a median of 34.8, SD: 14.4) and PP (mean of 35.2 years, a median of 37.0, SD: 13.7) samples (*p* = 2.0 × 10^−5^, paired sample *t* test, Fig. 4c). For the AK and cSCC analysis, we predicted the DNAm age of 12 normal epidermis samples, 16 AK epidermis samples, and 18 cSCC epidermis samples (data obtained from [24]). When comparing the DNAm age residuals from healthy skin controls (mean of 8.88 DNAm residuals, SD: 9.66) and AK (mean of − 6.82 DNAm residuals, SD: 23.8), we observed a statistically significant decrease in the molecular age of AK samples (*p* = 0.026, *t* test, Fig. 4d), which was not detected previously, when the same data were analyzed using the pan-tissue DNAm algorithm [24]. The epidermal stem cell origin of AK and cSCC [24] may justify the lower DNAm age detected by our algorithm.

**Fig. 5:**
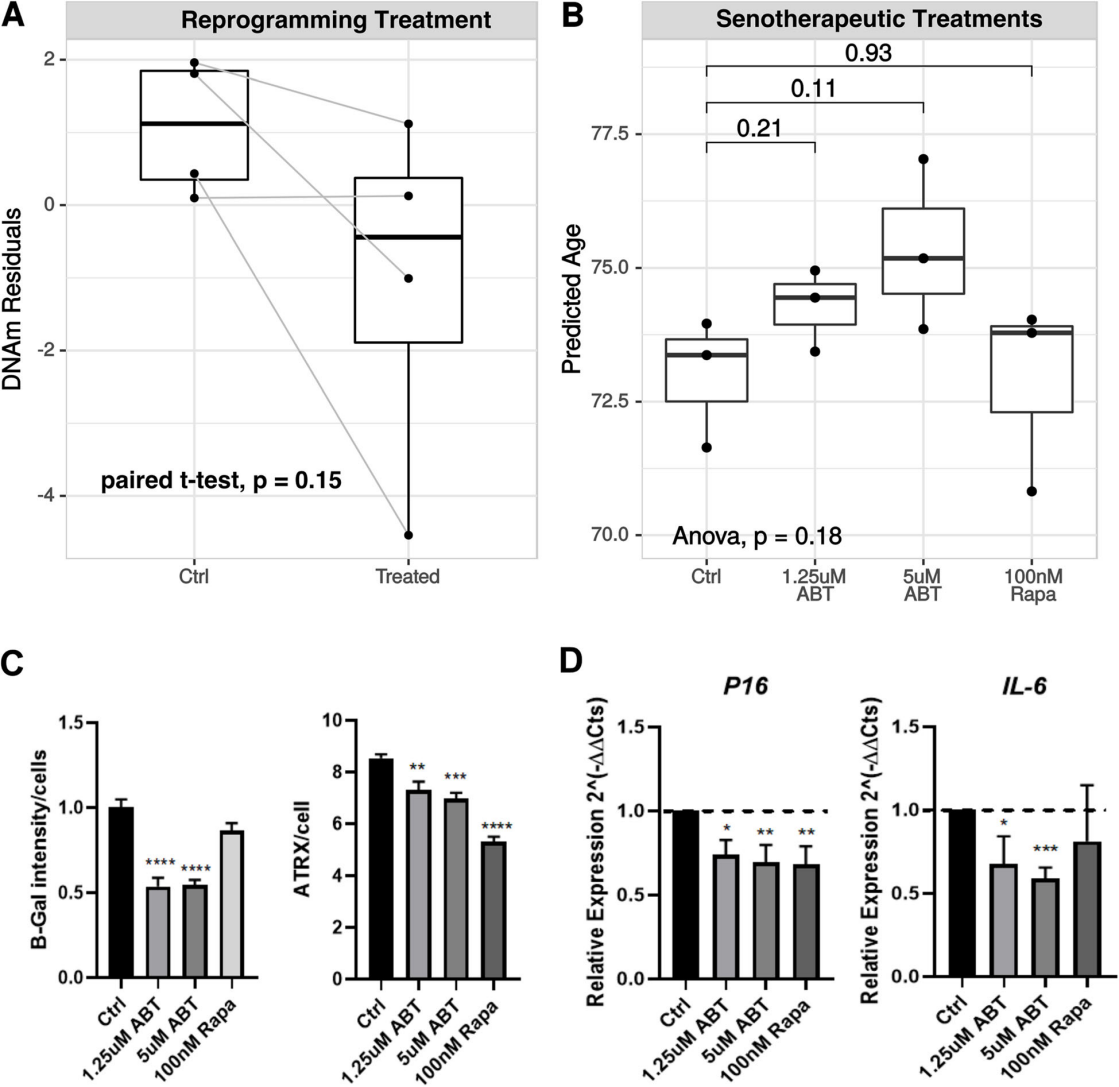
Skin-specific DNAm predictor as a tool to validate the senotherapeutic potential of different compounds. **a** DNAm age residuals of primary human dermal fibroblasts treated with OSKMLN reprogramming factors (GSE142439 data) and untreated control samples (Ctrl). **b**–**d** Primary human dermal fibroblasts derived from HGPS donor treated with ABT-263 (ABT) at 1.25 and 5 μM, as well as 100 nM of Rapamycin (Rapa) for 3 days. Untreated cells were considered as controls (Ctrl). **b** Predicted DNAm age using the new skin-specific molecular clock, **c** senescence-associated beta-galactosidase (SA-β-Gal) staining intensity per nuclei, and the number of ATRX foci/cell. **d** Relative gene expression of *CDKN2A* (P16) and *IL6* measured by qRT-PCR compared to untreated samples using ANOVA and Bonferroni *p < 0.05; **p < 0 < 0.01; ***p < 0.001; ****p < 0.0001

Next, we assessed the capacity of the skin-specific DNAm age predictor to validate the effects of numerous aging-related interventions. Recently, the treatment of cells with the reprogramming factors OCT4, SOX2, KLF4, c-MYC, LIN28, and NANOG (OSKMLN) [25] was shown to promote a partial reversion in cellular age without altering cellular identity. Here, we applied the skin-specific DNAm age predictor to analyze the published data and observe the effect of the reprogramming treatment on fibroblast DNAm age. No statistically significant difference was found (*p* = 0.15, paired sample *t* test, Fig. 5a) when comparing the DNAm age residuals from treated (mean age residuals of − 1.08, SD: 2.47) and control samples (mean age residuals of 1.08, SD: 0.95), likely due to the small dataset and the statistical test utilized.

When applying the skin-specific algorithm to evaluate known senotherapeutic treatments on human fibroblast cultures, we treated HGPS cells with ABT-263 and Rapamycin, drugs known to be senolytic and senomorphic respectively, i.e., drugs that specifically target cellular senescence, an altered cell state associated with aging and age-related diseases, and therefore expected to reduce cellular DNAm age [26, 27]. After 3-day treatments, no statistically significant differences were observed when comparing treated and untreated samples (Fig. 5b). Interestingly, despite the lack of statistical significance in the DNAm alteration of 2D cultured fibroblasts, detectable alterations in other phenotypes associated with aging were observed. Specifically, both ABT-263 treatments (1.25 μM and 5 μM) promoted a statistically significant reduction of approximately 50% in the mean senescence-associated beta-galactosidase (SA-β-Gal) staining intensity of treated samples (*p* < 0.0001), while Rapamycin (100 nM) treatment did not significantly alter the SA-β-Gal staining levels (Fig. 5c, left graph). The treatments with ABT-263 at 1.25 and 5 μM, as well as the treatment with Rapamycin at 100 nM, promoted a statistically significant reduction of *p* < 0.01, *p* < 0.001, and *p* < 0.0001, respectively, in the average number of ATRX foci/cell (Fig. 5c, right graph), which is an early predictor of senescence burden [28]. Such phenotypic alterations corroborated the statistically significant reduction of approximately 30% of the *CDKN2* (P16) mRNA expression detected in all experimental conditions (1.25 μM ABT-263 *p* < 0.05; 5 μM ABT-263 *p* < 0.01; Rapamycin 100 nM *p* < 0.01, compared to untreated samples—Fig. 5d left graph). The ABT-263-treated samples also presented a statistically significant decrease in *IL6* mRNA expression (ABT-263 1.25 μM, *p* < 0.05; ABT-263 5 μM, *p* < 0.001 compared to control, according to *t* test analysis—Fig. 5d, right graph). In all comparisons using 2D cell cultures, the previously published skin and blood DNAm estimator presented similar results to the estimator presented here (Additional File 11–Fig S7).

**Fig. 6:**
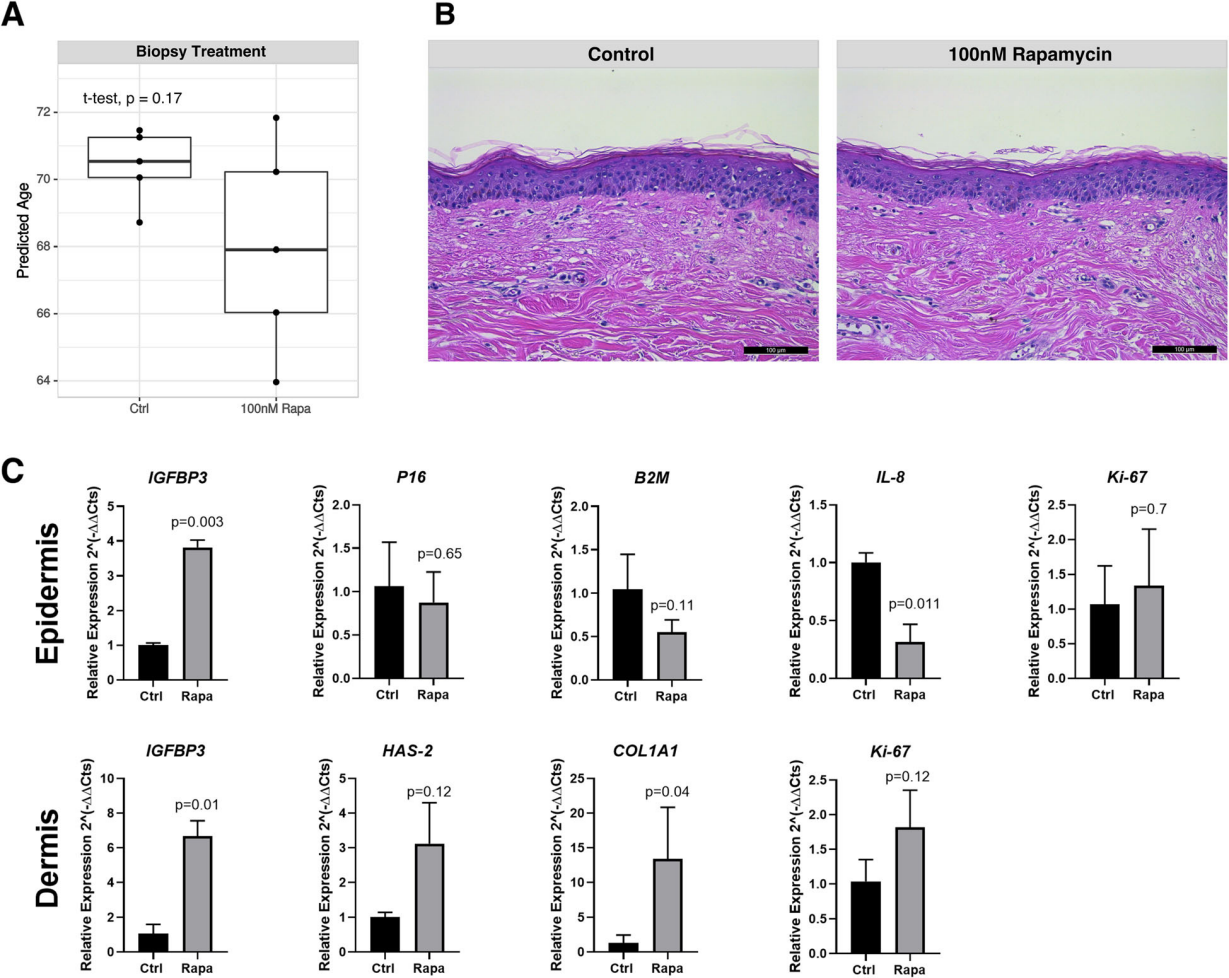
Effect of senotherapeutic treatments in human skin biopsies treated with 100 nM Rapamycin for 5 days. **a** Predicted DNAm age using the skin-specific molecular clock. **b** Representative images of H&E of treated and untreated (control) samples, **c** mRNA expression in the epidermis, and **d** mRNA expression in the dermis. *Ctrl* control, *IGFBP3* insulin growth factor binding protein 3, *B2M* β2 microglobulin, *IL8* interleukin-8, *HAS2* hyaluronic acid synthase 2, *COL1A1* collagen type 1 alpha 1 compared to untreated samples using ANOVA and Bonferroni, or *t* test. Data refer to experiments performed in triplicate

We then evaluated the ability of our DNAm estimator to predict treatment efficacy using fresh human skin biopsy samples, which were maintained in culture for 5 days. In this case, the skin-specific algorithm developed in the present study was able to detect a DNAm age reduction in biopsies after treatment with 100 nM Rapamycin (mean age of 70.4, SD of 1.10 for control versus mean age of 68.0, SD of 3.16 for treatment, *p* = 0.17 *t* test, Fig. 6a). Oppositely, the skin and blood DNAm age estimator predicted an increase of the molecular age after treatment (mean age of 69, SD of 3.95 for control versus mean age of 70.1, SD of 3.24 for treatment, *p* = 0.64, *t* test, Fig. S7g). The treatment with Rapamycin did not modify the overall morphological structure of the skin assessed by hematoxylin and eosin (H&E) staining (Fig. 6b), but it resulted in a statistically significant increase in insulin growth factor binding protein 3 (*IGFBP3*) mRNA expression and a significant decrease in interleukin-8 (*CXCL8 or* IL8) mRNA expression (Fig. 6c), similar to what has been published previously [29]. *CDKN2A* (P16), β2 microglobulin (*B2M*), and marker of proliferation Ki-67 (*MKi67*) presented non-statistically significant mRNA expression alterations. In the dermis, the decreased DNAm age of Rapamycin-treated samples was associated with a statistically significant increase in collagen type I alpha 1 (*COL1A1*) and *IGFBP3* mRNA expression, as well as non-significant increases in hyaluronic acid synthase-2 (*HAS2*) and *MKi67* mRNA expression (Fig. 6d).

## Discussion

DNA methylation molecular clocks constitute algorithms which highly correlate (*r* > 0.8) DNA methylation patterns of specific biological samples with chronological age or time [30]. Two of the first-reported clocks include the molecular algorithm built by Hannum et al., based on methylome data of blood samples obtained from 656 individuals [31] and the pan-tissue molecular algorithm built by Steve Horvath using 8000 samples of 51 different healthy tissues and cell types [2]. Since then, many other molecular clocks have been built, all presenting pros and cons. Of note, as knowledge regarding DNAm age accumulates, it becomes increasingly clear that tissue-specific algorithms are potentially more accurate than pan-tissue counterparts. Furthermore, it has recently been shown that epigenetic age, as calculated by DNAm algorithms, is not only influenced by chronological age but also by the health status of the sample [32]. Therefore, such algorithms are currently considered as possible tools to predict lifespan and healthspan [32]. In the present study, we proposed that such molecular clocks can also be used to accurately assess skin health, aging, and also the effect of experimental interventions on skin DNAm age.

Even though the causes of the accumulation of epigenetic alterations in DNA are unknown, current knowledge points to the participation of both intrinsic (intracellular) and extrinsic (extracellular) processes. The skin is no different from other tissues in this sense, as its aging reflects both intrinsic and extrinsic processes, such as genetic and metabolic factors, as well as UV exposure and the general exposure to the greater environment [33]. As observed in other tissues, skin aging is generally accompanied by the accumulation of senescent cells [34, 35], reduced cellular proliferation/tissue renewal, and altered extracellular matrix, which are associated with skin wrinkling, sagging, altered pigmentation [36], and cancer [37]. Still, accumulating evidence shows that tissues age differently, both in the sense that aging may be desynchronized within a single multicellular organism, and also that different methylation markers may be more relevant to calculate DNAm age from one tissue than another. Those aspects of tissue aging became evident in the Horvath pan-tissue molecular clock algorithm, which determined that hormone exposed organs age more quickly than those that are not exposed [38], and also presented unequal performances when calculating the age of different tissues [2]. Of note, the pan-tissue molecular clock presented high error rates when used to calculate the age of skin samples and cells, especially fibroblasts [2].

In recognition of such limitations, the same author recently developed another molecular clock algorithm, the skin and blood molecular clock [19]. Such an algorithm was built using methylome data of human fibroblasts, keratinocytes, buccal cells, endothelial cells, lymphoblastoid cells, skin, blood, and saliva samples, leading to improved performance compared to previous data for DNAm age prediction in skin samples [19]. In the present study, we tested both algorithms pan-tissue as well as the skin and blood and compared them to the algorithm we built using DNA methylation data of human skin biopsies only. Such a strategy is exclusive in the sense that it is the first molecular clock developed from DNA methylation data of human skin biopsies, and, interestingly, it led to compelling results. The focus on skin biopsy samples limited the amount of data used to build the algorithm but resulted in a highly efficient DNAm age predictor specific for human skin. Furthermore, the algorithm could be executed with high accuracy and a smaller standard deviation compared to former DNAm age predictors. The algorithm developed here also accurately predicted the DNAm age of fibroblasts, which was significantly influenced by donor age and cell passage, as previously reported by others [11, 12].

The new skin-specific algorithm developed in the present study is based on the analysis of 2266 CpG sites, of which less than 3% overlap with the skin and blood algorithm developed previously [19]. As mentioned, a link between cause and consequence has not yet been established between the methylation pattern of the skin during aging and the resulting DNAm age. Nevertheless, the analysis of the biological function attributed to the genome regions assessed by the top 300 probes has led to interesting insights. In the skin, the genes located in the regions interrogated by the probes are related to several cellular processes. The most enriched pathway in this analysis was cAMP signaling, which has previously been associated with organismal aging and cellular senescence [39].

When analyzing the DNAm data obtained from donors of different age groups (< 30 years old, between 30 and 60 years old, and > 60 years old), we noticed the predictors used in the skin-specific DNAm algorithm detected that, while young methylomes were more similar among each other, old methylomes appeared to be substantially more heterogeneous. This is in consonance to published observations, which indicate that while methylation patterning within an individual becomes more homogeneous with age, the differences between individuals increase [16].

The skin-specific DNAm predictor could also detect the reduction of DNAm age of primary human dermal fibroblasts with a partial pluripotency reprogramming regimen, corroborating previous reports [25]. Surprisingly, the treatment of progeroid fibroblasts with the senolytic ABT-263, although promoting a decrease in the number of senescent cells and the gene expression of aging markers, displayed a trend of increasing the DNAm age. This data reinforces that alterations in gene expression and DNAm age may highlight markers of aging with different sensitivity, and that mRNA expression does not necessarily correlate with methylation alterations. Short-term treatment with Rapamycin also altered the gene expression of age markers but did not alter the DNAm age, despite revealing that the treatment promoted a trend in DNAm age decrease. When the same data was analyzed using the skin and blood DNAm algorithm, Rapamycin treatment also failed to promote statistically significant DNAm age alteration but resulted in a trend of DNAm age increase, which conflicts with the decrease in mRNA markers related to aging and senescence. Moreover, it suggests that increased treatment times should be investigated to validate the effect of senotherapeutics on the DNAm age.

Using human skin biopsies, we noticed that Rapamycin promoted a nonsignificant decrease in the calculated DNAm age of treated samples. Nevertheless, alterations at the gene expression level were observed with the significant increase in *IGFBP3* mRNA expression and also a significant decrease in *CXCL8* (*IL8*) mRNA expression, similar to what has been published previously [29]. In the dermis, the decreased DNAm age of Rapamycin-treated samples was associated with a statistically significant increase in *COL1A1* and *IGFBP3* mRNA expression, suggesting DNAm age is related to several established markers of skin aging.

The skin maintains a few characteristics which render this organ as an interesting target for DNAm age studies. First, it is one of the few organs from which it is relatively easy to obtain samples for analysis. It can also be replicated in vitro with high fidelity; it is the first organ to externalize signs of aging, and suffers extreme external influence from environmental stimuli such as the sun and pollution. Interestingly, in addition to its particularities and the appeal of cosmetic applications, due to its size, the skin can play a major role in organismal inflammation levels and has been linked to numerous chronic diseases of aging [40]. Hu et al. recently demonstrated that epidermal dysfunction largely accounts for age-associated elevations in circulating cytokine levels and that improving epidermal function reduced these levels in mice [41]. Ye et al. provided similar evidence in humans [40]. In the last 5 years, studies of psoriasis [6] and dermatitis [7] have similarly determined that skin inflammation likely increases the risk of cardiovascular disease. Therefore, the development of a skin-specific DNAm age predictor can offer a powerful tool for the comprehension of skin diseases and how it may influence overall organismal health status and DNAm age. Since skin cancer has been correlated to age [37], DNAm analysis may also benefit skin cancer studies.

Prior to the present study, DNAm age predictors failed to detect any influence of AK with DNAm age [23], discouraging any correlation between skin aging and AK. In the present study, we were able to detect significant differences between the DNAm age of healthy and AK samples as well as significant differences in psoriatic and cSCC samples. Nevertheless, our observation of a decrease in the mDNA age of psoriatic samples compared to normal counterparts disagrees with previously published data describing that psoriatic lesions are characterized by the presence of senescent keratinocytes, mainly observed in the mid and upper epidermal layers [42]. The keratinocytes derived from psoriatic diseases have already been shown to express high levels of *CDKN2* (*P16*), *CDKN1A* (*P21*), as well as low *CDK1* and *CCNA1*, in addition to IGFBP2 [42], which is a component of SASP [43]. Still, the development of a skin-specific DNAm age predictor may offer powerful means for the comprehension of skin illnesses.

The application of senotherapeutic molecules for skin treatment is a very recent concept, which has very limited clinical evidence of efficacy currently. In our hands, the importance of executing several technical replicates for each DNAm analysis was important, due to the high standard deviation observed between samples. In a study involving a small number of patients, the topical application of Rapamycin for 6 months promoted a reduction in p16^INK4A^ protein levels in the skin and also improved skin appearance [29]. Here, we tested short Rapamycin treatment in skin biopsies and could note a reduction in DNAm age, as well as mRNA expression of age-related genes. This data supports the use of DNAm age as a parameter to be investigated during the research and validation of novel senotherapeutic models for skin care as well as interventions that modulate skin aging. The minimum amount of time required to promote significant changes in the skin mDNA may widely vary according to the intervention executed. Therefore, this remains to be validated in larger and long-term studies.

## Conclusions

Taken together, the present data suggests that in vitro models recapitulate key aspects of skin aging and DNAm age analysis using the present algorithm provides a more comprehensive and highly accurate method to analyze human skin health status and aging. Such data may support further investigation and understanding of how skin ages, as well as how DNAm age of the skin is impacted by health status and experimental treatments.

## Methods

### Methylation data access and pre-processing

Pre-processed beta values from GSE51954, GSE90124, GSE73894, and GSE142439 datasets were individually downloaded using the getGEO function from “GEOquery” R library version 2.54.1 [44], while E-MTAB-4385 and E-MTAB-5738 raw files were downloaded from ArrayExpress and converted to beta values using the “minfi” package version 1.32.0 [45]. Datasets used for algorithm construction were individually processed, merged, and normalized using the function betaqn from the “wateRmelon” package version 1.30.0 [46], which performs quantile normalization on the beta values. We have checked for batch effects when combining the three dataset for the algorithm construction. Even though the removal of potential batch effects in our data using the comBat function from the sva package version 3.34.0 [44, 47] seems to improve data homogenization, the effect was the opposite in the final model when predicting unseen data. Therefore, we did not remove the batch effect to avoid the overfitting of the final model. Heatmaps were constructed using the pheatmap package version 1.0.12 using beta values, z-scaled across samples. Probes (rows) were clustered using Pearson correlation and samples were ordered based on their chronological age.

### Age prediction analysis with previously published molecular clocks

Quantile-normalized beta values for all samples were used as input for both R software codes underlying the pan-tissue [2] and/or skin and blood [19]. The R codes were retrieved from the paper’s supplementary information. For datasets where chronological age was informed, we also calculated the DNAm age residual, defined as the residual of a linear model where the independent variable is chronological age and the response is DNAm age. Box plots were used to compare age predictions among groups in different datasets.

### RNA-Seq data access and pre-processing

SRA files from the project SRP082426 [48] were downloaded and converted to fastq files using SRA Toolkit version 2.8.2-1. Trimmed reads using the software Trimmomatic version 0.37 [49] with default options were mapped to the human genome (GRCh38–ENSEMBL release 88) using STAR version 2.5.3a [50] with default parameters for single unstranded reads as per developer’s manual. Htseq-count version 0.11.1 [51] was used to assign uniquely mapped reads to genes (excluding pseudogenes) according to the annotation in the Homo_sapiens.GRCh38.89.gtf (ENSEMBL release 88). Read counts were analyzed using the R package DESeq2 version 1.26.0 [52] and libraries were normalized using the estimateSizeFactors function of the package after samples were split into three groups according to their chronological age: age 1 (20 samples from donors under 30 years old); age 2 (20 samples from donors between 30 and 60 years old), and age 3 (51 samples from donors above 60 years old). Heat maps were constructed using the pheatmap package version 1.0.12 using a regularized Log2-transformed counts-per-million, z-scaled across samples. Genes (rows) were clustered using Pearson correlation and samples were ordered based on their chronological age. Box plots using ggplot2 package version 3.3.1 [53] were used to compare gene expression among age groups.

### Pathway enrichment analysis

Genes associated with the probes according to the Illumina manifest were retrieved from a list of probes positively and negatively correlated with age in our model. The gene lists were then analyzed for known biological functions or processes enriched using the over-representation analysis (ORA) methodology in the WEB-based GEne SeT AnaLysis Toolkit [54] using the Kyoto Encyclopedia of Genes and Genomes (KEGG) database. Genes associated with the top 300 probes ranked according to their importance for the model were also analyzed by this methodology. *P* values were controlled for false discovery rate (FDR) using the Bonferroni method.

### Feature selection

Cross-reactive probes [20], probes on the sex chromosomes, probes that were not present in the Infinium MethylationEPIC Array (Illumina), and probes with missing values were excluded. In order to reduce the data dimensionality, the R package “FeatureSelection” version 1.0.0 (https://github.com/mlampros/FeatureSelection) was used, which is a wrapper to select features based on three different algorithmic implementations proper for high-dimensional data sets: Glmnet is a package that fits a generalized linear model via penalized maximum likelihood; Xgboost stands for “Extreme Gradient Boosting” and is a fast implementation of the well-known boosted trees; ranger is a fast implementation of random forest, particularly suited for high-dimensional data. In this step, highly correlated features were removed.

### Machine learning training

Five ML algorithms implemented in the R package caret version 6.0-86 [55] were used with the training dataset: the ranger implementation of random forest (using 100 trees); support vector machines with radial basis function kernel; ridge regression, which penalizes sum of squared coefficients (L2 penalty); Lasso regression, which penalizes the sum of absolute values of the coefficients (L1 penalty); and elastic net, a convex combination of ridge and Lasso. In each case, 50-fold resampling cross-validation was used for the optimization of the tuning parameters. Model prediction errors were computed using mean absolute error (MAE) and/or root mean squared error (RMSE). Fitness levels and significance of the applied regression models were evaluated by computing Pearson’s correlation coefficient using the training data. RMSE was used to select the optimal model using the smallest value. We also compared the performance of the select model with the pan-tissue and skin and blood DNAm age predictors by using the same parameters in a novel dataset consisting of 16 skin biopsies samples from different donors whose methylation levels were accessed by the Infinium MethylationEPIC Array (Illumina).

### Cell culture and treatments

Primary human dermal fibroblasts derived from an HGPS donor were obtained from The Progeria Research Foundation Cell and Tissue Bank and cultured in Dulbecco’s modified Eagle medium (Invitrogen), supplemented with 10% *v*/*v* fetal bovine serum (FBS; Invitrogen) and 1% *v*/*v* Penicillin-Streptomycin (Invitrogen). Before reaching complete confluence, cells were expanded using 0.25% Trypsin/EDTA (Gibco), followed by inactivation of the enzyme using FBS-containing medium. Primary human dermal fibroblasts derived from healthy donors were purchased from Coriell Institute for Medical Research and maintained in the same conditions.

ABT-263 (ApexBio, final concentration: 1.25 or 5 μM) or Rapamycin (Fisher Scientific, final concentration 100 nM) were added to cell culture media of fibroblasts for 2D assays and maintained for 3 days. Samples were collected for analysis after three days of recovery following ABT-263 or Rapamycin removal.

### β-galactosidase staining

β-galactosidase staining was performed using Senescence β-Galactosidase Staining Kit (Cell Signaling, 9860S), following the manufacturer’s instructions. Cells were washed with 1× PBS and fixed for 10–15 min. Then, cells were washed 2 times with 1× PBS and incubated with β-Galactosidase Staining Solution overnight at 37 °C in a dry incubator without CO_2_. Cells were then stained with DAPI and observed by × 10 magnification (6D High Throughput, Nikon) and blue staining quantified as the mean color intensity compared to the total number of cells using CellProfiler^TM^.

### ATRX staining

ATRX foci were detected by immunofluorescence, using ATRX (Santa Cruz Biotechnology, D5 - sc55584) antibody diluted 1:2000, followed by goat anti-mouse IgG H&L-Alexa Fluor® 488 (Abcam, Cambridge, MA, ab150113), as described previously [28]. Briefly, cells were fixed with 4% paraformaldehyde solution for 10 min. Permeabilization was performed for 5 min with 0.1% Triton followed by blocking for 40 min with 0.5% Tween and 1% BSA. The primary antibody was incubated overnight at 4 °C. After three washes with PBS, cells were incubated at room temperature for 1 h with the secondary antibody + Hoechst 33342. Cells were imaged at × 40 magnification using the IN Cell Analyzer 2500 (GE Healthcare). The analysis was performed using the IN Cell Developer toolbox. The average ATRX foci per cell was defined by the total ATRX foci/total nuclei. A minimum of 150 cells was analyzed per experimental condition.

### Ex-vivo skin samples and Treatment with Rapamycin

Skin samples from a healthy donor (Female, Caucasian, 79 years) were obtained from ZenBio (Research Triangle, NC) and maintained in an air-liquid interface in Dulbecco’s modified Eagle medium (Invitrogen, Carlsbad, CA), supplemented with 10% *v*/*v* FBS. The skin samples were treated with either vehicle or 100 nM Rapamycin (Fisher Scientific, Hampton, NH) in the media, on days 1 and 3. After 5 days, the samples were harvested and fixed in formalin for histology, or used for RNA and DNA isolation.

### RNA isolation and RT-qPCR

RNA was isolated from skin biopsies or cell culture samples using the Quick RNA Miniprep kit (Zymo Research, Irvine, CA) and following the manufacturer instructions. Total RNA was then quantified and 1 μg was used for reverse transcription, using the high-capacity cDNA Reverse Transcription Kit (Thermo Fisher Scientific). qPCR was performed using PerfeCTa® qPCR ToughMix®, Low ROX™(QuantaBio) and the Taqman (Invitrogen) probes for *CDKN2* (P16) (Hs00923894_m1), *IL6* (hS00174131_m1), *IGFBP3* (Hs00181211_m1), *B2M* (Hs00187842_m1), *CXCL8* (IL8) (Hs00174103_m1), *MKi67* (*Ki67*) (Hs04260396_g1), *HAS2* (Hs00193435_m1), *COL1A1* (Hs00164004_m1), and *GAPDH* (Hs02758991_g1).

### DNA sample acquisition and methylation analysis

Total DNA samples were obtained from cultured cells or human skin biopsy samples (purchased from Genoskin, Inc., France) using the QIAamp DNA Mini Kit (QIAGEN) and following manufacturer instructions. DNA methylation assessment was performed by The University of British Columbia (Vancouver, Canada) using the human Illumina Infinium EPIC 850K chip. DNA samples are described in the Table S1 and include 16 skin biopsy samples, 3 samples of technical replicates of HGPS passage 11, 3 samples of technical replicates of HGPS passage 19, 3 samples of technical replicates of human primary fibroblasts obtained from 29-year-old donor, 3 samples of technical replicates of human primary fibroblasts obtained from the 84-year-old donor, 3 samples of technical replicates of fibroblasts treated with 100 nM Rapamycin, 1.25 5 μM ABT-263, or non-treated, 5 skin biopsy samples which were considered as untreated controls, and 5 skin biopsy samples treated with 100 nM Rapamycin. The raw image data was processed using the commands preprocessRaw() followed by preprocessSWAN(). Methylation signals (*M* values) were then converted to ratios using the ratioConvert() and next to beta values using getBeta(), all functions implemented in the “minfi” R package version 1.32.0 [45]. Beta values were normalized using the betaqn() method, which quantile normalizes betas, implemented by the “wateRmelon” package version 1.30.0 [46]. Normalized beta values were used for age estimation.

### Statistical analysis

Data was tested for normal distribution performing the Shapiro-Wilk test. In cases where more than 2 groups were compared, we performed one-way ANOVA followed by Bonferroni’s multiple comparisons test. For cases where paired samples were provided, a paired *t* test was performed. *P* ≤ 0.05 were considered statistically significant. Analyses were performed using the program GraphPad Prism 8 software or R version 3.6.3 software.

### Skin-specific DNAm predictor

The algorithm described here is available to the scientific committee through an Application Programming Interface (API) that can be accessed by the link: www.molclock.com. As an input, users will upload their matrix containing pre-processed and normalized methylation levels (beta values) measured on the Illumina BeadChip platform and will receive as output a table with the predicted DNAm age.

## Supplementary information

**Additional file 1.** Supplementary Figure 1. Experimental design. (A) Schematic representation of the workflow used for constructing and validating the skin-specific DNAm age estimator. (B) Schematic representation of the skin-specific molecular clock applications envisioned in this work.

**Additional file 2.** Supplementary Table 1. Description of all data used in this work.

**Additional file 3.** Supplementary Figure 2: Normalization quality control. Density plots showing the methylation beta values distribution before and after normalizing all three datasets (E_MATB_4385, GSE51954, and GSE90124) by quantile.

**Additional file 4.** Supplementary Figure 3: Pre-selected probes for algorithm training. Top 50 probes ranked according to different algorithms implementations in the feature selection step. Probes that were cross-reactive, targeting sex chromosomes or were not present in the current version of EPIC array were excluded, as described in the methodology section.

**Additional file 5.** Supplementary Figure 4. Age distribution of samples in training and testing datasets. Samples were randomly distributed between training and testing datasets, following a balanced distribution according to donor age.

**Additional file 6 **Supplementary Figure 5. Comparative analysis of machine learning algorithm performance. Machine Learning (ML) algorithms random forest (rf), support vector machines (svm), lasso, elastic net (enet) and ridge were compared according to their performance, as assessed by Mean Absolute Error (MAE), Root mean squared error (RMSE) and maximum *R*^2^ (R Squared).

**Additional file 7.** Supplementary Table 2. Age estimations comparison among three DNAm age predictors for 16 biopsies samples (external validation dataset).

**Additional file 8.** Supplementary Table 3. The enrichment method Over Representation Analysis (ORA) was performed using the Kyoto Encyclopedia of Genes and Genomes (KEGG) database. p values were corrected to control for FDR using the Bonferroni method and only tests with p < 0.05 were considered significant.

**Additional file 9.** Supplementary Figure 6. Probes shared by different DNAm age predictors. (A) Analysis of common probes among the Skin-Specific, the Skin & Blood, and the Pan-Tissue DNAm age predictors. (B) Heat map of DNA methylation levels of the eight probes shared by the three DNAm algorithms. Color codes represent beta DNAm values after row-wise z-score transformation. Probes (rows) are ordered according to their importance. Samples were ordered according to their age. (C) Expression among age groups of genes associated with the shared probes in B.

**Additional file 10.** Supplementary Table 4. List of probes shared by the Skin-Specific, the Skin & Blood (H2), and the Pan-Tissue (H1) DNAm age predictors.

**Additional file 11.** Supplementary Figure 7. DNAm age estimation using the Skin & Blood algorithm. Similar to the analysis performed with the Skin-Specific DNAm age predictor, the Skin & Blood DNAm estimator was used to calculate the DNAm age of (A) primary human dermal fibroblasts obtained from healthy donors from increasing chronological age. (B) DNAm age of primary human dermal fibroblasts derived from an HGPS donor increased with the cell passage. (C) DNAm age of human psoriatic (PP) and paired uninvolved psoriatic (PN) skin tissues (GSE73894). (D) DNAm age residuals of normal epidermis tissues, AK samples, and cSCC epidermis samples (E-MTAB-5738 data). (E) DNAm age residuals of primary human dermal fibroblasts treated with OSKMLN reprogramming factors and controls (Ctrl) (GSE142439 data). (F) DNAm age of primary human dermal fibroblasts derived from HGPS donor treated with ABT-263 (ABT) at 1.25 and 5 μM, as well as 100 nM of Rapamycin (Rapa) for three days. Untreated cells were considered as controls (Ctrl). (G) DNAm age of human skin biopsies treated with 100 nM Rapamycin (Rapa) for five days and untreated controls (Ctrl).

## Data Availability

The five datasets produced for this work have been uploaded to the GEO database under the accession number GSE151617 (reference Series).

## Abbreviations

ABT: ABT-263
SA-BGal: Senescence-associated beta-galactosidase staining
DNAm: DNA methylation
FDR: False discovery rate
HGPS: Hutchinson-Gilford Progeria
H&E: Hematoxylin and eosin
KEGG: Kyoto Encyclopedia of Genes and Genomes
MAE: Mean absolute error
ML: Machine learning
ORA: Over representation analysis
PP: Psoriatic skin tissue
PN: Control non-psoriatic skin tissue
Rapa: Rapamycin
RMSE: Root mean squared error
SASP: Senescence-associated secretory phenotype
SD: Standard deviation
UC: normal skin tissues
