# Supplementary figures and images for: Highly accurate skin-specific methylome analysis algorithm as a platform to screen and validate therapeutics for healthy aging
Source: Clin Epigenetics. 2020 Jul 13;12:105. doi: 10.1186/s13148-020-00899-1 (PMC7359467; doi:10.1186/s13148-020-00899-1)

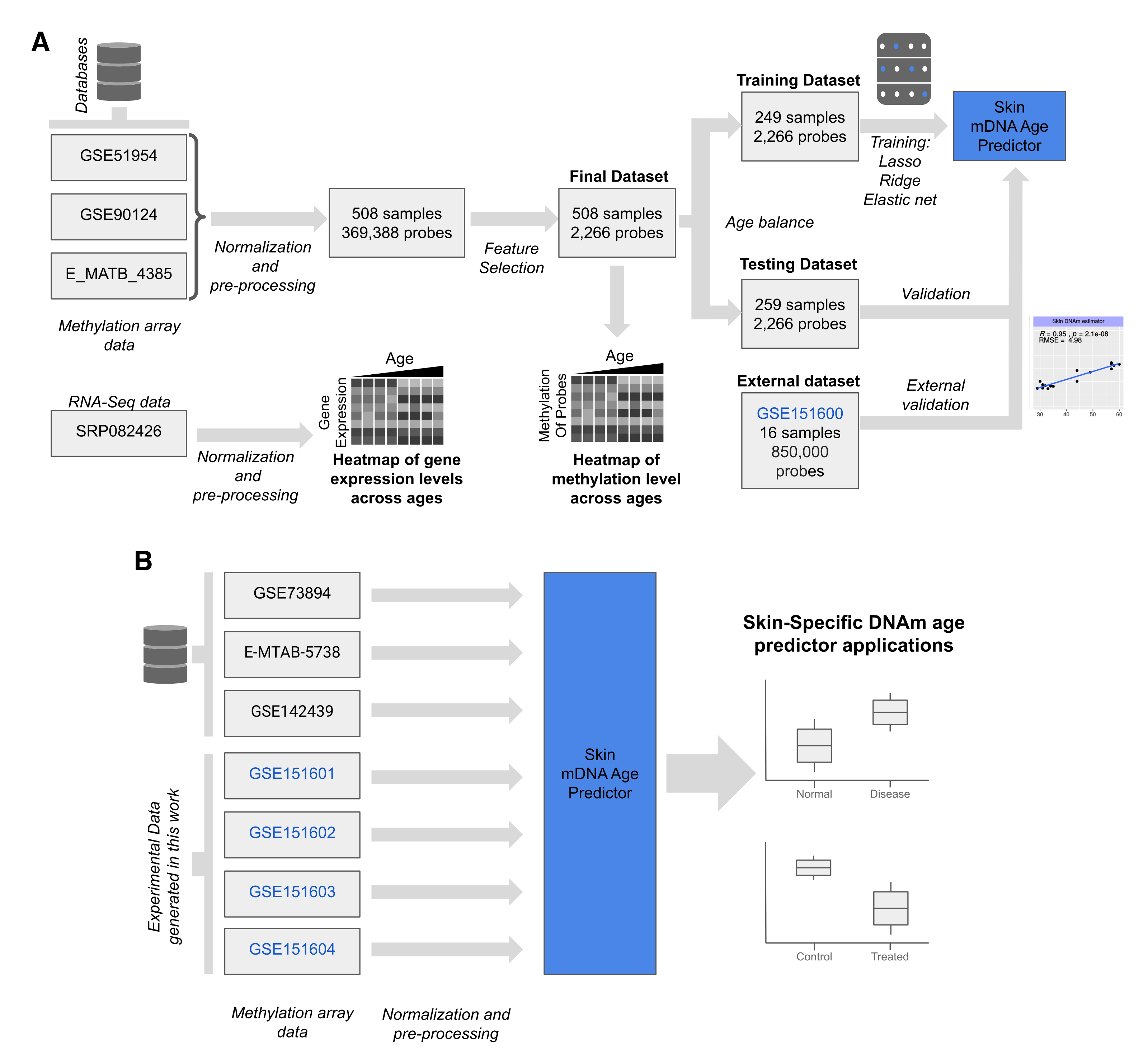

Supplement: Supplementary file 1 — Additional file 1. Supplementary Figure 1. Experimental design. (A) Schematic representation of the workflow used for constructing and validating the skin-specific DNAm age estimator. (B) Schematic representation of the skin-specific molecular clock applications envisioned in this work. [file 13148_2020_899_MOESM1_ESM.png]

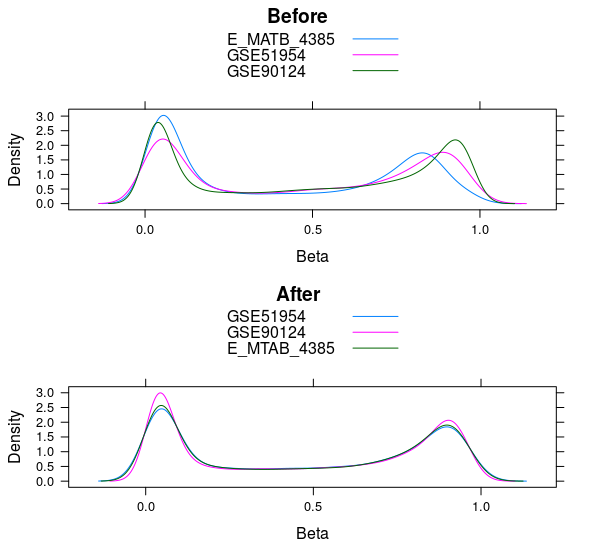

Supplement: Supplementary file 3 — Additional file 3. Supplementary Figure 2: Normalization quality control. Density plots showing the methylation beta values distribution before and after normalizing all three datasets (E_MATB_4385, GSE51954, and GSE90124) by quantile. [file 13148_2020_899_MOESM3_ESM.png]

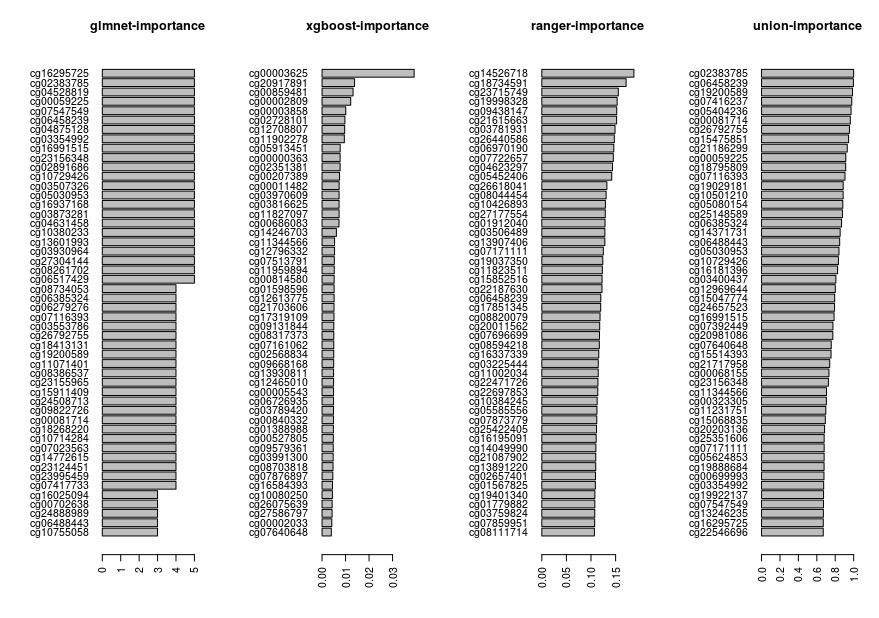

Supplement: Supplementary file 4 — Additional file 4. Supplementary Figure 3: Pre-selected probes for algorithm training. Top 50 probes ranked according to different algorithms implementations in the feature selection step. Probes that were cross-reactive, targeting sex chromosomes or were not present in the current version of EPIC array were excluded, as described in the methodology section. [file 13148_2020_899_MOESM4_ESM.jpeg]

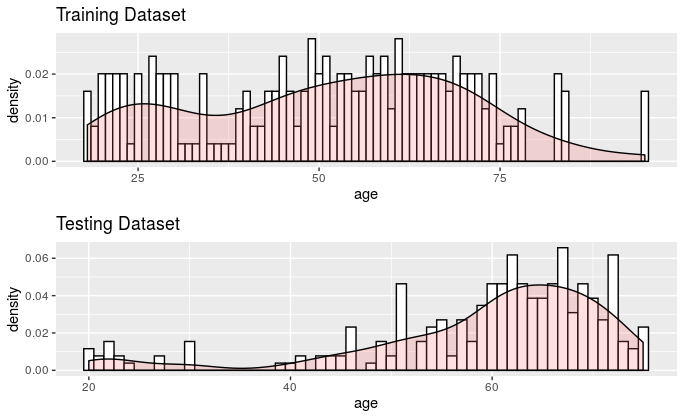

Supplement: Supplementary file 5 — Additional file 5. Supplementary Figure 4. Age distribution of samples in training and testing datasets. Samples were randomly distributed between training and testing datasets, following a balanced distribution according to donor age. [file 13148_2020_899_MOESM5_ESM.png]

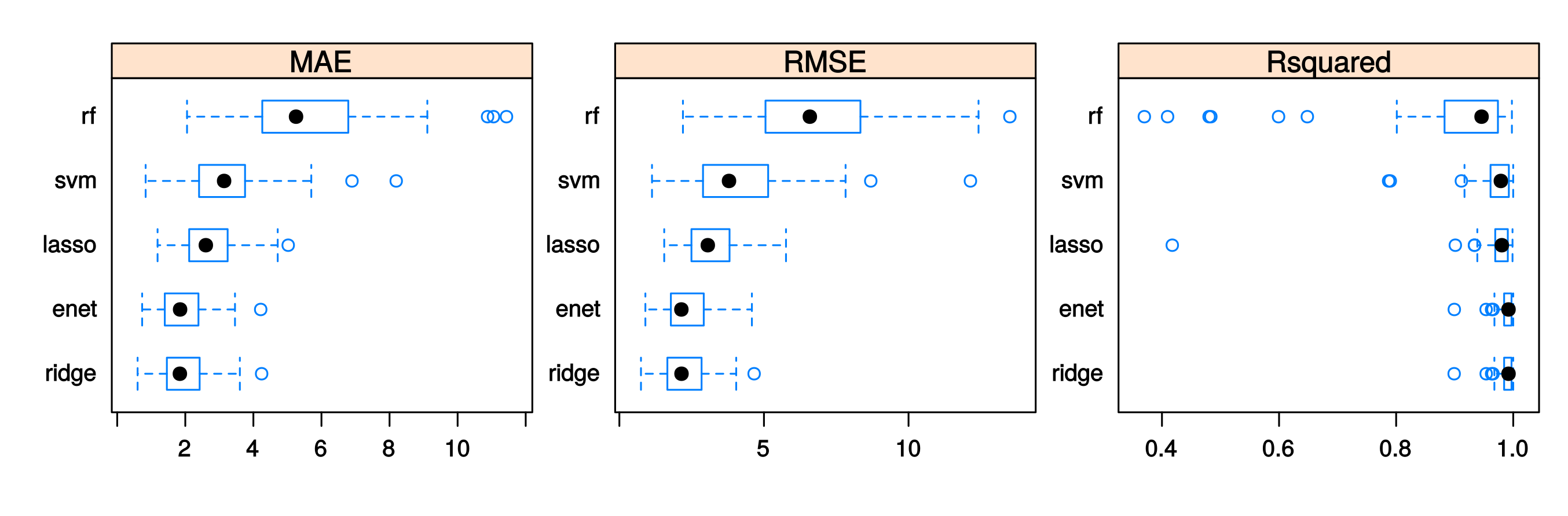

Supplement: Supplementary file 6 — Additional file 6 Supplementary Figure 5. Comparative analysis of machine learning algorithm performance. Machine Learning (ML) algorithms random forest (rf), support vector machines (svm), lasso, elastic net (enet) and ridge were compared according to their performance, as assessed by Mean Absolute Error (MAE), Root mean squared error (RMSE) and maximum R2 (R Squared). [file 13148_2020_899_MOESM6_ESM.png]

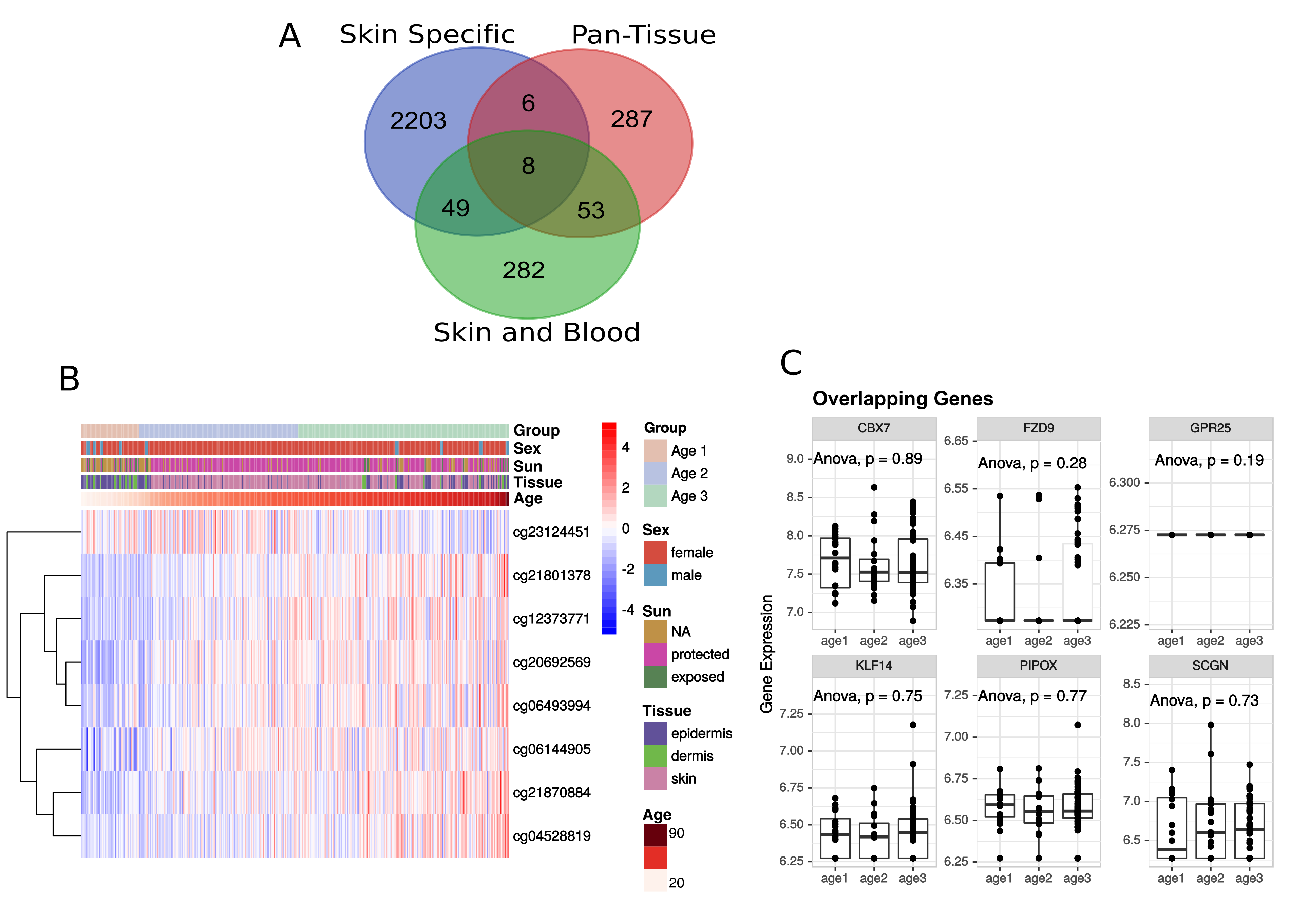

Supplement: Supplementary file 9 — Additional file 9. Supplementary Figure 6. Probes shared by different DNAm age predictors. (A) Analysis of common probes among the Skin-Specific, the Skin & Blood, and the Pan-Tissue DNAm age predictors. (B) Heat map of DNA methylation levels of the eight probes shared by the three DNAm algorithms. Color codes represent beta DNAm values after row-wise z-score transformation. Probes (rows) are ordered according to their importance. Samples were ordered according to their age. (C) Expression among age groups of genes associated with the shared probes in B. [file 13148_2020_899_MOESM9_ESM.png]

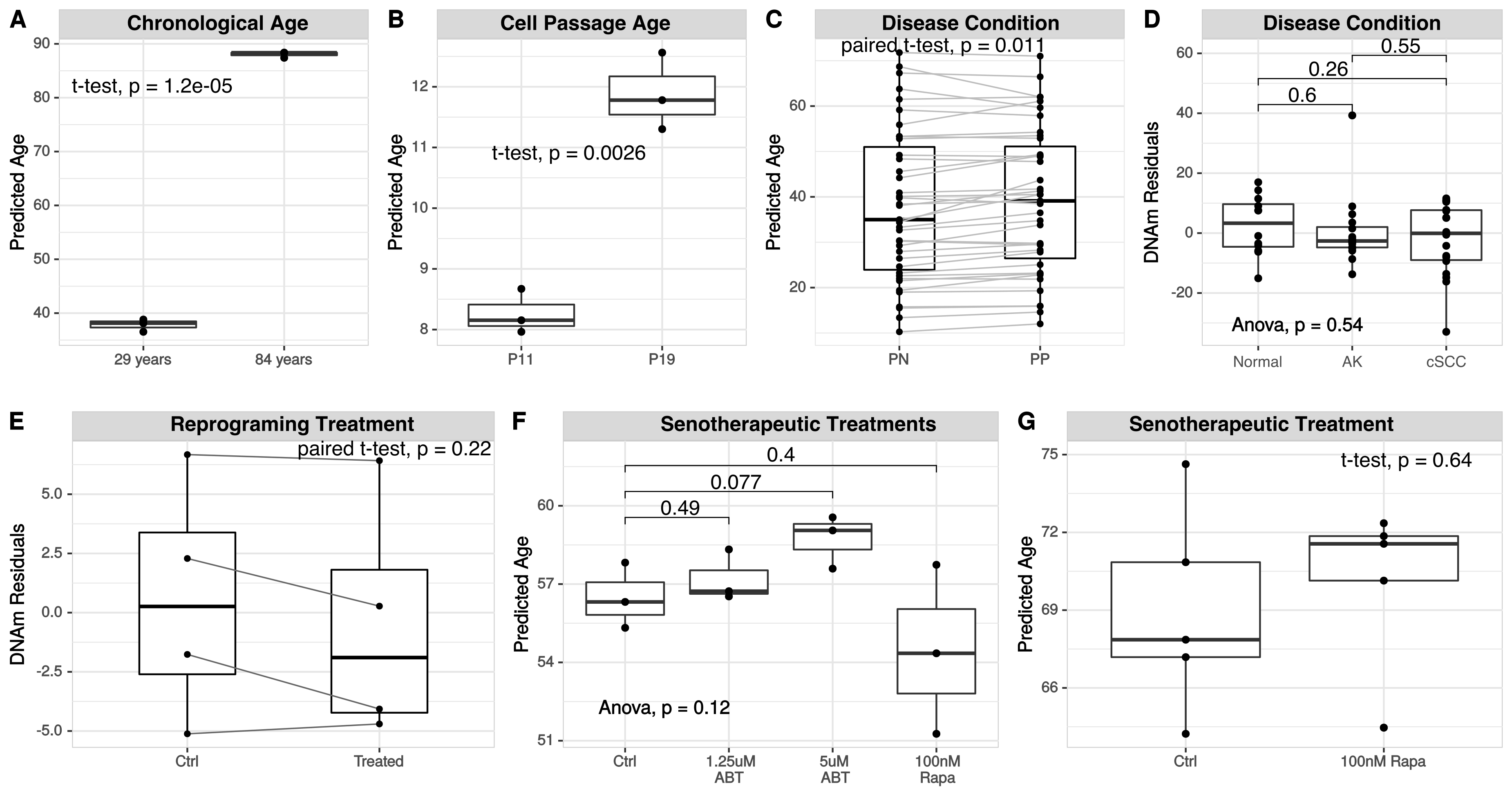

Supplement: Supplementary file 11 — Additional file 11. Supplementary Figure 7. DNAm age estimation using the Skin & Blood algorithm. Similar to the analysis performed with the Skin-Specific DNAm age predictor, the Skin & Blood DNAm estimator was used to calculate the DNAm age of (A) primary human dermal fibroblasts obtained from healthy donors from increasing chronological age. (B) DNAm age of primary human dermal fibroblasts derived from an HGPS donor increased with the cell passage. (C) DNAm age of human psoriatic (PP) and paired uninvolved psoriatic (PN) skin tissues (GSE73894). (D) DNAm age residuals of normal epidermis tissues, AK samples, and cSCC epidermis samples (E-MTAB-5738 data). (E) DNAm age residuals of primary human dermal fibroblasts treated with OSKMLN reprogramming factors and controls (Ctrl) (GSE142439 data). (F) DNAm age of primary human dermal fibroblasts derived from HGPS donor treated with ABT-263 (ABT) at 1.25 and 5 μM, as well as 100 nM of Rapamycin (Rapa) for three days. Untreated cells were considered as controls (Ctrl). (G) DNAm age of human skin biopsies treated with 100 nM Rapamycin (Rapa) for five days and untreated controls (Ctrl). [file 13148_2020_899_MOESM11_ESM.png]
