# Supplementary material for: Highly accurate skin-specific methylome analysis algorithm as a platform to screen and validate therapeutics for healthy aging
Source: Clin Epigenetics. 2020 Jul 13;12:105. doi: 10.1186/s13148-020-00899-1 (PMC7359467; doi:10.1186/s13148-020-00899-1)
Supplement: Supplementary file 2 — Additional file 2. Supplementary Table 1. Description of all data used in this work. [file 13148_2020_899_MOESM2_ESM.docx]

**Supplementary Table 1 - Description of all data used in this work.**

| **Dataset ID** | **Origin** | **Number of samples** | **Type of sample** | **Sex** | **Ethnicity** | **Donor Age** | **Platform** | **Analysis** | **Figure** |
| --- | --- | --- | --- | --- | --- | --- | --- | --- | --- |
| GSE51954 | GEO | 78 | 40 dermis  38 epidermis | 43 F  35 M | Caucasian | 20-95 | Illumina Infinium HumanMethylation450 BeadChip | -Skin DNA age Predictor -Heatmap of methylation level across ages | -Fig 1A and 1B -Fig 2A, 3C and S6B |
| GSE90124 | GEO | 322 | whole skin | 322 F | Caucasian | 39-83 | Illumina Infinium HumanMethylation450 BeadChip |  |  |
| E_MATB_4385 | ArrayExpress | 108 | epidermis | 108 F | Caucasian | 18-78 | Illumina Infinium HumanMethylation450 BeadChip |  |  |
| GSE151600 | OneSkin | 16 | whole skin | 16 F | Caucasian | 29-60 | Illumina Infinium HumanMethylation850 BeadChip | -Algorithm external validation | Fig 1C |
| SRP082426 | SRA | 91 | whole skin | 91 F | Caucasian |  | Illumina HiSeq 2500 | -Heatmap of gene expression levels across ages | -Fig 2B, 3B and S6C |
| GSE151601 | OneSkin | 6 | 3 dermal fibroblasts from HGPS at passage 11 and 3 at passage 19 | 6 F | Caucasian | 6y11m | Illumina Infinium HumanMethylation850 BeadChip | -DNAm Age predictions | -Fig 4A -Fig S7A |
| GSE151602 | OneSkin | 6 | Human primary dermal fibroblasts | 6 F | Caucasian | 29 and 84 | Illumina Infinium HumanMethylation850 BeadChip |  | -Fig 4B -Fig S7B |
| GSE73894 | GEO | 78 | 39 human psoriatic - PP and 39 paired uninvolved psoriatic - PN skin tissues | 15 F 24 M | Asian | NA | Illumina Infinium HumanMethylation450 BeadChip |  | -Fig 4C -Fig S7C |
| E-MTAB-5738 | ArrayExpress | 46 | 12 normal epidermis samples, 16 AK - actinic keratosis and 18 cSCC - cutaneous squamous cell carcinoma samples | 45 M 1 F | Caucasian |  | Illumina Infinium HumanMethylation850 BeadChip |  | -Fig 4D -Fig S7D |
| GSE142439 | GEO | 8 | 4 skin primary fibroblast control and 4 after reprogramming treatment | 3 M 1 F | Caucasian | 61-69 | Illumina Infinium HumanMethylation850 BeadChip |  | -Fig 5A -Fig S7E |
| GSE151603 | OneSkin | 12 | 3 samples of technical replicates of dermal fibroblasts from HGPS donor treated with 100 nM Rapamycin, 1.25 and 5 μM ABT-263, or non-treated | 12 F | Caucasian | 6y11m | Illumina Infinium HumanMethylation850 BeadChip |  | -Fig 5B -Fig S7F |
| GSE151604 | OneSkin | 10 | 5 skin biopsy samples untreated and 5 skin biopsy samples treated with 100 nM Rapamycin | 10 F | Caucasian | 79y | Illumina Infinium HumanMethylation850 BeadChip |  | -Fig 6A -Fig S7G |
