# Supplementary material for: Highly accurate skin-specific methylome analysis algorithm as a platform to screen and validate therapeutics for healthy aging
Source: Clin Epigenetics. 2020 Jul 13;12:105. doi: 10.1186/s13148-020-00899-1 (PMC7359467; doi:10.1186/s13148-020-00899-1)
Supplement: Supplementary file 7 — Additional file 7. Supplementary Table 2. Age estimations comparison among three DNAm age predictors for 16 biopsies samples (external validation dataset). [file 13148_2020_899_MOESM7_ESM.docx]

**Supplementary Table 2 - Performance of DNAm age predictor algorithms.** Age estimations comparison among three DNAm age predictors for 16 biopsies samples (external validation dataset).

| **Sample_ID** | **Sex** | **Age** | **Skin- Specific DNAm age predictor** | **Delta** | **Pan- tissue DNAm age estimator** | **Delta** | **Skin & blood DNAm age estimator** | **Delta** |
| --- | --- | --- | --- | --- | --- | --- | --- | --- |
| 18-0173 | Female | 29 | 34.5 | 5.5 | 21.1 | -7.9 | 34.8 | 5.8 |
| 18-0053 | Female | 30 | 40.0 | 10.0 | 20.9 | -9.1 | 43 | 13 |
| 18-0081b | Female | 31 | 34.6 | 3.6 | 22.5 | -8.5 | 40.6 | 9.6 |
| 18-0172 | Female | 31 | 37.4 | 6.4 | 22.4 | -8.6 | 43.2 | 12.2 |
| 18-0149b | Female | 32 | 36.6 | 4.6 | 26.2 | -5.8 | 42.5 | 10.5 |
| 18-0158 | Female | 33 | 34.2 | 1.2 | 21.3 | -11.7 | 41.9 | 8.9 |
| 18-0098b | Female | 34 | 36.2 | 2.2 | 27.3 | -6.7 | 45.8 | 11.8 |
| 18-0079b | Female | 35 | 36.1 | 1.1 | 29.4 | -5.6 | 43.1 | 8.1 |
| 18-0147 | Female | 44 | 40.0 | -4.0 | 27.1 | -16.9 | 46.1 | 2.1 |
| 18-0159 | Female | 44 | 48.5 | 4.5 | 30.3 | -13.7 | 48.4 | 4.4 |
| 18-0148 | Female | 49 | 47.2 | -1.8 | 35.3 | -13.7 | 56.2 | 7.2 |
| 18-0080b | Female | 57 | 54.6 | -2.4 | 36.1 | -20.9 | 59.3 | 2.3 |
| 18-0117b | Female | 57 | 53.8 | -3.2 | 36.5 | -20.5 | 57.8 | 0.8 |
| 18-0171b | Female | 57 | 49.7 | -7.3 | 30.3 | -26.7 | 57.2 | 0.2 |
| 18-0140 | Female | 58 | 52.3 | -5.7 | 33.3 | -24.7 | 57 | -1 |
| 18-0193 | Female | 60 | 53.4 | -6.6 | 35.8 | -24.2 | 56.3 | -3.7 |
