# Supplementary material for: Highly accurate skin-specific methylome analysis algorithm as a platform to screen and validate therapeutics for healthy aging
Source: Clin Epigenetics. 2020 Jul 13;12:105. doi: 10.1186/s13148-020-00899-1 (PMC7359467; doi:10.1186/s13148-020-00899-1)
Supplement: Supplementary file 8 — Additional file 8. Supplementary Table 3. The enrichment method Over Representation Analysis (ORA) was performed using the Kyoto Encyclopedia of Genes and Genomes (KEGG) database. p values were corrected to control for FDR using the Bonferroni method and only tests with p < 0.05 were considered significant. [file 13148_2020_899_MOESM8_ESM.docx]

**Supplementary Table 3 - Signaling pathways enriched for genes associated with probes positively correlated within the Skin-Specific DNAm age predictor**. The enrichment method Over Representation Analysis (ORA) was performed using the Kyoto Encyclopedia of Genes and Genomes (KEGG) database. p values were corrected to control for FDR using the Bonferroni method and only tests with p < 0.05 were considered significant.

| **Description** | **Enrichment Ratio** | **p-value** | **FDR** | **Genes** |
| --- | --- | --- | --- | --- |
| Calcium signaling pathway | 3.1 | 0.00002 | 0.00495 | *CACNA1G; CCKBR ; HTR7; GRIN2A; ADRB1; PTGFR; TBXA2R; CACNA1C; SPHK2; CACNA1I; RYR2; CACNA1S; GRIN2D; CAMK2D;*  *AVPR1A; CACNA1H; PRKCG; NOS1* |
| Cushing syndrome | 3.24686 | 0.00003 | 0.00495 | *CDK4; LEF1; CACNA1G; WNT10B; ATF4; CACNA1C; FZD10; CACNA1I; CACNA1S; FZD9; KCNA4; CDKN2B; CAMK2D; NR5A1; CRHR1; CACNA1H* |
| Circadian entrainment | 3.07190 | 0.00005 | 0.00548 | *GRIA2; CACNA1G; GRIN2A; CACNA1C; CACNA1I; RYR2; GRIN2D; CAMK2D;*  *GNG7; CACNA1H; PRKCG; NOS1* |
| Alanine, aspartate and glutamate metabolism | 1.11996 | 0.00010 | 0.00801 | *GPT2; GOT1; ADSSL1; ABAT; IL4I1; ALDH4A1; GAD1* |
| Neuroactive ligand-receptor interaction | 8.86370 | 0.00054 | 0.03499 | *GRIK5; THRB; GRIA2; CCKBR; ADRA2A; HTR7; GABRB3; GRIN2A; GRM2; GHSR; ADRB1; PTGFR; TBXA2R; GLRA1; LEP; PRLHR; GRIN2D; DRD4; CRHR1; AVPR1A* |
| Cocaine addiction | 1.56795 | 0.00086 | 0.04284 | *GRIA2; CDK5R1; BDNF; ATF4; GRIN2A; GRM2; GRIN2D* |
| Cortisol synthesis and secretion | 2.04793 | 0.00092 | 0.04284 | *CACNA1G; ATF4; CACNA1C; CACNA1I; CACNA1S; KCNA4; NR5A1; CACNA1H* |
