# Supplementary material for: Highly accurate skin-specific methylome analysis algorithm as a platform to screen and validate therapeutics for healthy aging
Source: Clin Epigenetics. 2020 Jul 13;12:105. doi: 10.1186/s13148-020-00899-1 (PMC7359467; doi:10.1186/s13148-020-00899-1)
Supplement: Supplementary file 10 — Additional file 10. Supplementary Table 4. List of probes shared by the Skin-Specific, the Skin & Blood (H2), and the Pan-Tissue (H1) DNAm age predictors. [file 13148_2020_899_MOESM10_ESM.docx]

**Supplementary Table 4 - List of probes shared by the Skin-Specific, the Skin & Blood (H2), and the Pan-Tissue (H1) DNAm age predictors.**

| **Probes** | **chr** | **pos** | **strand** | **UCSC RefGene Name** | **UCSC RefGene Group** |
| --- | --- | --- | --- | --- | --- |
| cg23124451 | chr22 | 39548131 | + | CBX7 | Body |
| cg21870884 | chr1 | 200842429 | - | GPR25 | 1stExon |
| cg20692569 | chr7 | 72848481 | + | FZD9 | 1stExon |
| cg06144905 | chr17 | 27369780 | + | PIPOX | TSS200 |
| cg06493994 | chr6 | 25652602 | - | SCGN;SCGN | 1stExon;5'UTR |
| cg12373771 | chr22 | 17601381 | - | CECR6;CECR6 | 1stExon;5'UTR |
| cg21801378 | chr15 | 72612125 | - | BRUNOL6 | 1stExon |
| cg04528819 | chr7 | 130418315 | - | KLF14 | 1stExon |
